# Fecal microbiota transplantation from duodenal light stimulation–conditioned donors is associated with improved glucose tolerance and intestinal incretin-related remodeling in diabetic Goto–Kakizaki rats

**DOI:** 10.3389/fcimb.2026.1900693

**Published:** 2026-09-11

**Authors:** Jisong Ahn, Minhong Kim, Se Hee Min, Hyun-Gyeong Bae, Ji-Yun Bae, Jinhee Kwon, Hana Yi, Do Hyun Park

**Affiliations:** 1 Division of Gastroenterology, Department of Internal Medicine, University of Ulsan College of Medicine, Asan Medical Center, Seoul, Republic of Korea; 2 Integrated Biomedical and Life Science, Graduate School, Korea University, Seoul, Republic of Korea; 3 Interdisciplinary Program in Precision Public Health, Korea University, Seoul, Republic of Korea; 4 Division of Endocrinology and Metabolism, Department of Internal Medicine, University of Ulsan College of Medicine, Asan Medical Center, Seoul, Republic of Korea; 5 Department of Medical Science, Asan Medical Institute of Convergence Science and Technology, University of Ulsan College of Medicine, Asan Medical Center, Seoul, Republic of Korea; 6 Healthcare Technology Examination Division, Digital Convergence Examination Bureau, Korean Intellectual Property Office (KIPO), Daejeon, Republic of Korea

**Keywords:** duodenal light stimulation, fecal microbiota transplantation, gut microbiota, incretin, shotgun metagenomics, type 2 diabetes mellitus

## Abstract

**Background:**

Type 2 diabetes mellitus (T2DM) is a complex metabolic disorder characterized by impaired glucose homeostasis and β-cell dysfunction. Emerging evidence suggests that the gut microbiota–incretin axis may contribute to metabolic regulation. However, whether microbiota from duodenal light stimulation (DLS)-conditioned donors can influence metabolic phenotypes via fecal microbiota transplantation (FMT) remains unclear.

**Methods:**

We evaluated whether FMT from DLS-conditioned donors was associated with metabolic and intestinal changes in diabetic GK (Goto–Kakizaki) rats. Recipients received FMT from week −2 to week 0 and were then followed for 7 weeks after the final FMT dose. Metabolic phenotyping, intestinal histology, short-chain fatty acid (SCFA) profiling, and shotgun metagenomic profiling of bacterial and viral communities were performed.

**Results:**

FMT recipients showed within-group improvement in OGTT glucose profiles, without significant changes in fasting glucose levels. Total glucose AUC0–120 was significantly reduced within the FMT group in the within-group (period) comparison; however, the group × period interaction was not significant, indicating no statistically significant longitudinal between-group treatment effect. Early GLP-1 responses showed a modest increasing trend in FMT recipients, whereas total GLP-1 AUC0–120 was not significantly changed. HOMA-β (homeostatic model assessment of β-cell function) increased within the FMT group, and pancreatic insulin-positive area was greater in FMT recipients than in controls at the study endpoint. Intestinal remodeling was evident, including an increased villus:crypt ratio and increased colonic GLP-1–positive cells. FMT was associated with fecal bacteriome differences, including one FDR-significant taxon and several nominally associated taxa, such as *Akkermansia muciniphila* and *Xylanibacter rodentium*. No significant global shift in fecal virome composition was observed, although selected viral taxa showed nominal group-associated differences that did not remain significant after FDR correction. Exploratory network analysis suggested group-specific bacteriome–virome association patterns after FMT.

**Conclusion:**

FMT from DLS-conditioned donors was associated with improved glucose tolerance, intestinal incretin-related remodeling, increased pancreatic insulin-positive area, and fecal bacteriome differences in diabetic GK rats based on within-group longitudinal changes for the glucose- and β-cell-related outcomes, for which the group × period interactions were not significant, whereas the histological and microbiome differences reflect cross-sectional between-group comparisons at the study endpoint. These findings support a hypothesis-generating link between donor-conditioned FMT, intestinal remodeling, and microbiome-associated metabolic regulation, while further studies are required to define DLS-specific and donor-derived effects.

## 1 Introduction

Type 2 diabetes mellitus (T2DM) is a chronic progressive metabolic disorder characterized by insulin resistance and a gradual decline in pancreatic β-cell mass and function (Kahn, 2003; Cnop et al., 2005). The global prevalence of diabetes is projected to increase substantially, from 537 million adults in 2021 to 783 million by 2045, and impose a growing clinical and socioeconomic burden (Magliano and Boyko, 2021). Progressive β-cell dysfunction, driven by metabolic stressors such as oxidative stress, inflammation, and endoplasmic reticulum stress, is a central feature of disease progression, highlighting the need for therapeutic strategies that preserve β-cell function in addition to improving glycemic control (Cnop et al., 2005; Dludla et al., 2023).

Glucagon-like peptide-1 (GLP-1), an incretin hormone secreted by intestinal L cells, plays a critical role in glucose homeostasis by enhancing glucose-dependent insulin secretion and supporting β-cell function and survival (Drucker, 2003; Brubaker and Drucker, 2004). Beyond glycemic regulation, GLP-1 also influences gastric emptying, appetite, and cardiometabolic outcomes (Silver et al., 2023). Although GLP-1–based pharmacotherapies have demonstrated significant clinical efficacy, their use is constrained by gastrointestinal side effects, high cost, and the need for repeated administration (Amori et al., 2007; Hamed et al., 2024; Wennberg et al., 2025). These limitations have motivated interest in alternative strategies that modulate endogenous incretin pathways in a more sustainable and physiologically integrated manner.

Accumulating evidence suggests that the gut microbiota is an important regulator of host metabolism and incretin signaling (Heiss and Olofsson, 2018; Zeng et al., 2024). Microbial communities influence nutrient sensing, enteroendocrine function, and intestinal barrier integrity, thereby contributing to metabolic homeostasis (Duca et al., 2021; Dmytriv et al., 2024). In particular, alterations in gut microbiota composition have been associated with impaired GLP-1 secretion and glucose metabolism in T2DM, supporting the concept that microbiota-targeted interventions may offer therapeutic potential (Greiner and Bäckhed, 2011; Utzschneider et al., 2016; Wang et al., 2020).

The duodenum represents a key interface between nutrient exposure, microbial signals, and enteroendocrine regulation (Theodorakis et al., 2006; Ma et al., 2012). In this context, we previously demonstrated that duodenal light stimulation (DLS) using dual wavelengths (630 and 850 nm) improved glucose metabolism in Goto–Kakizaki (GK) rats, a non-obese model of T2DM (Min et al., 2022). These effects were accompanied by increased circulating GLP-1 levels, enhanced β-cell insulin expression, and remodeling of the gut microbial community. These findings suggest that DLS may influence host metabolism, with microbiota-associated changes representing one potential component of this response. Device-based donor conditioning may provide an experimental strategy to generate donor fecal material after a defined localized intestinal intervention, thereby allowing evaluation of whether an intervention-modified donor intestinal environment is associated with recipient phenotypes after FMT.

In the present study, we investigated whether fecal microbiota transplantation (FMT) from DLS-conditioned donors was associated with metabolic and intestinal changes in diabetic recipient GK rats. Specifically, we examined OGTT glucose profiles, circulating GLP-1 responses, colonic GLP-1–positive cells, intestinal structure, and pancreatic insulin-positive β-cell area. In addition, we performed integrated bacteriome and virome analyses to characterize FMT-associated fecal microbial profiles and explore their relationships with host metabolic parameters. Through this approach, we aimed to evaluate whether FMT from DLS-conditioned donors is associated with host metabolic phenotypes and microbiome-related changes in T2DM.

## 2 Materials and methods

### 2.1 Animals and experimental design

Seven-week-old male GK rats were obtained from SLC (Shizuoka, Japan). Animals were housed individually under controlled conditions (22 ± 2 °C, 55 ± 10% humidity, 12-h light/dark cycle, 150–300 lux) with *ad libitum* access to standard chow and water. Body weight and food intake were recorded weekly. Recipient rats were randomly assigned to either the control group receiving saline (CTL) or the FMT group (n = 7 per group). Recipient rats received FMT or saline from week −2 to week 0 by oral gavage every other day for 2 weeks (seven administrations in total), each preceded by overnight fasting. After completion of FMT or saline administration, recipient rats were followed for an additional 7 weeks. Metabolic phenotyping was performed before FMT initiation at Pre-Tx (pre-treatment) and 7 weeks after FMT completion at Post-Tx (post-treatment). Fasting blood glucose was measured after overnight fasting, whereas non-fasting blood glucose was measured under *ad libitum*-fed conditions in tail-vein blood. During oral glucose tolerance tests (OGTTs), tail-vein blood samples were collected for the measurement of blood glucose, plasma insulin, and plasma GLP-1. At the study endpoint, 7 weeks after FMT completion, animals were fasted overnight and euthanized under isoflurane anesthesia (2–3%). Blood was centrifuged at 3,000 rpm for 15 min at 4 °C to obtain plasma, which was stored at −80 °C until further analysis. Tissue samples, including pancreas, duodenum, and colon, were collected and either snap-frozen in liquid nitrogen or fixed in 4% paraformaldehyde for histological analyses. For donor experiments, a separate cohort of GK rats underwent DLS. Donor fecal samples collected during weeks 2–3 post-DLS were used for FMT, and separate donor fecal aliquots were used for downstream analyses: fecal samples collected at week 2 post-DLS were used for shotgun metagenomic sequencing (n = 5 donors), whereas fecal samples collected before DLS and at week 3 post-DLS were used for short-chain fatty acid profiling (n = 5 donors). All animal procedures were approved by the Institutional Animal Care and Use Committee of Asan Medical Center (IACUC; approval no. 2023-20-227) and were conducted in accordance with national guidelines.

### 2.2 Donor conditioning and fecal microbiota transplantation

Donor rats were treated using the same dual-wavelength LED protocol (630 + 850 nm) previously described for the 630 + 850 nm group (Min et al., 2022). A custom-built LED device incorporating 630- and 850-nm LED chips was used for the DLS experiments. The device was configured as a catheter-based system for duodenal application. Briefly, the LED catheter consisted of mini-LED chips mounted along a 50-mm illuminated segment of a polyurethane catheter. The catheter was 2.5 mm in diameter and 6 cm in length, and the LED chips were arranged at 5-mm intervals in a four-surface configuration to improve circumferential illumination of the proximal duodenum. For the dual-wavelength protocol, 630- and 850-nm irradiation was applied for 500 s. The 630-nm component was delivered at 0.036 W, with an irradiance of 3.7 mW/cm² and an energy density of 1.85 J/cm², whereas the 850-nm component was delivered at 0.046 W, with an irradiance of 7.7 mW/cm² and an energy density of 3.85 J/cm². Under anesthesia, the LED catheter was inserted into the proximal duodenum through laparotomy- and gastrostomy-guided placement, and the proximal duodenal segment was irradiated. During DLS, surface temperature was monitored using infrared thermal imaging to assess potential thermal effects. Representative schematic, device, intraoperative, and thermal images are provided in **Supplementary Figure 1**.

FMT was performed using fecal material obtained from DLS-conditioned donor GK rats. Donor fecal samples were collected immediately before DLS and at weeks 2 and 3 after DLS, and samples from weeks 2–3 were used for FMT preparation. Samples were stored at −80 °C immediately after collection, thawed on the day of transplantation, and then processed for FMT preparation. Fecal material was suspended in sterile phosphate-buffered saline (PBS) at 1 g feces per 10 mL PBS, homogenized, and filtered through a 70-μm cell strainer (Corning, Cat. No. 431751, Corning, NY, USA) to remove large particulate debris. Recipient GK rats received approximately 1 mL of the freshly prepared fecal suspension per dose by oral gavage after overnight fasting. FMT was administered every other day for 2 weeks (seven administrations in total). Control animals received an equivalent volume of sterile saline under identical conditions. To characterize donor microbial metabolite profiles, fecal samples collected immediately before DLS and at week 3 after DLS were subjected to short-chain fatty acid (SCFA) analysis. SCFAs, including acetate, propionate, and butyrate, were quantified to assess donor microbial metabolite profiles. For donor bacteriome profiling, fecal samples collected at week 2 after DLS were subjected to shotgun metagenomic sequencing, and bacterial community composition was analyzed at the species level.

### 2.3 Oral glucose tolerance test

OGTTs were performed after an overnight fast of 12 h. Rats received an oral glucose load of 2.5 g/kg body weight by gavage. In recipient GK rats, blood samples were collected from the tail vein at 0, 30, 60, 90, and 120 min after glucose administration. In donor GK rats, blood glucose was measured at 0, 15, 30, 60, 90, and 120 min during OGTT. Blood glucose levels were measured immediately using a calibrated glucometer (Accu-Chek Instant, Roche Diagnostics, Mannheim, Germany). In donor GK rats, OGTTs were performed at baseline before DLS and at weeks 1 and 4 after DLS. In recipient GK rats, OGTTs were performed before FMT initiation at Pre-Tx and 7 weeks after FMT completion at Post-Tx. Glucose excursion curves were generated from these time-course measurements. Total glucose exposure during OGTT was quantified as the area under the curve from 0 to 120 min (AUC_0–120_), calculated using the trapezoidal method. Early glucose exposure was additionally quantified as glucose AUC0–60 in recipient GK rats, calculated from values at 0, 30, and 60 min.

### 2.4 Plasma hormone analysis

Plasma samples obtained from tail-vein blood collected during OGTTs were used for the measurement of insulin and GLP-1. Blood samples were collected into EDTA-coated tubes, immediately placed on ice, and centrifuged at 3,000 rpm for 15 min at 4 °C. Plasma samples were stored at −80 °C until analysis. Plasma insulin concentrations were measured with a Rat Insulin ELISA kit (Mercodia, Uppsala, Sweden), and plasma total GLP-1 concentrations were measured with a Rat GLP-1 ELISA kit (EZGLP1T-36K, Millipore, Burlington, MA, USA), each according to the manufacturer’s instructions. All samples were analyzed in duplicate. Time-course curves for insulin and GLP-1 in recipient GK rats were generated from measurements obtained at 0, 30, 60, and 120 min during OGTT. Insulin and GLP-1 AUC0–120 values were calculated using the trapezoidal method based on the available sampling points at 0, 30, 60, and 120 min, without interpolation. Early hormonal exposure was additionally quantified as insulin AUC0–60 and GLP-1 AUC0–60, calculated from values at 0, 30, and 60 min.

### 2.5 Assessment of β-cell function

Estimated β-cell function was assessed using the homeostasis model assessment of β-cell function (HOMA-β), calculated from fasting glucose and fasting insulin levels with the following equation:


HOMA−β = (360 × fasting insulin [μU/mL])/(fasting glucose [mg/dL] − 63)

Fasting blood samples were obtained after an overnight fast at Pre-Tx before FMT initiation and at Post-Tx, 7 weeks after FMT completion. Changes in HOMA-β were used to evaluate longitudinal alterations in estimated β-cell function within each group and between the CTL and FMT groups.

### 2.6 Histological analysis

At the study endpoint (Post-Tx, week 7), tissue samples from the pancreas, duodenum, and colon were collected immediately after euthanasia and fixed in 4% paraformaldehyde for 24–48 h. Fixed tissues were dehydrated through a graded ethanol series, cleared in xylene, and embedded in paraffin. Paraffin blocks were sectioned at a thickness of 4 μm and mounted on glass slides. Histological and immunohistochemical staining were performed according to standard protocols. All stained slides were digitized with a VS200 slide scanner (Olympus, Tokyo, Japan). A high-power field (HPF) was defined as 487 × 487 μm (0.237 mm^2^). All quantitative analyses were performed in a blinded manner.

#### 2.6.1 Pancreatic insulin immunohistochemistry

Immunohistochemical staining for insulin was performed on paraffin-embedded pancreatic sections with a Ventana Benchmark XT Autostainer (Roche, Basel, Switzerland) and the ultraView Universal DAB Detection Kit (760-500, Roche, Basel, Switzerland). Sections were incubated with a primary antibody against insulin (sc-8033, Santa Cruz Biotechnology, CA, USA; 1:2,000) followed by detection with the corresponding secondary system (760-4313, Roche, Basel, Switzerland). Whole-slide images were acquired using the VS200 slide scanner. The insulin-positive β-cell area was quantified and expressed as a percentage of total pancreatic tissue area using digital image analysis (Adobe Photoshop, Adobe Systems, San Jose, CA, USA). Analyses were performed for n = 7 animals per group.

#### 2.6.2 Colonic GLP-1 immunohistochemistry

Paraffin-embedded colonic sections were immunohistochemically stained to detect GLP-1–positive enteroendocrine cells. After antigen retrieval, sections were incubated with a rabbit monoclonal anti–GLP-1 antibody (ab226036, Abcam, Cambridge, UK; 1:250) followed by HRP-conjugated secondary antibody and DAB chromogen development. Digitized images were obtained using the VS200 slide scanner. For quantification, GLP-1–positive cells were manually counted in five non-overlapping HPFs per animal, and the counts were normalized to HPF area (0.237 mm^2^). Analyses were performed for n = 7 animals per group.

#### 2.6.3 Duodenal histology and morphometric analysis

Duodenal sections were stained with hematoxylin and eosin (H&E) to evaluate mucosal architecture. After standard deparaffinization and staining procedures, slides were digitized with the VS200 slide scanner. Quantitative morphometric analysis was performed using HALO image analysis software (Indica Labs, NM, USA). The following parameters were measured: villus height, crypt depth, villus-to-crypt ratio, and mucosal thickness. Measurements were obtained from well-oriented mucosal structures and averaged per animal. For group comparisons, the mean value per animal was used as a single representative data point. Analyses were performed for n = 7 animals per group.

#### 2.6.4 Colonic periodic acid–Schiff staining

Colonic sections were stained with PAS to assess mucin-containing structures. Slides were digitized using the VS200 slide scanner.

For quantitative analysis, the mucin-positive area was measured from five non-overlapping HPFs per animal using ImageJ software (National Institutes of Health, Bethesda, MD, USA) and expressed as a percentage of HPF area. One HPF corresponded to 487 × 487 μm (0.237 mm^2^). Owing to sample availability, PAS quantification was performed in a subset of animals (n = 5 per group).

### 2.7 Fecal short-chain fatty acid analysis

Fecal SCFAs were measured in both donor and recipient samples. For donor characterization, fecal samples collected immediately before DLS and at week 3 post-DLS were analyzed (n = 5 donors). For recipient analysis, fecal samples collected at Post-Tx (week 7) were analyzed to compare SCFA levels between CTL and FMT groups (n = 7 per group). SCFAs were extracted by homogenizing 0.2 g of feces in 1 mL distilled water, followed by centrifugation at 13,000 rpm for 10 min at 4 °C. The supernatant (150 μL) was transferred to a headspace vial and mixed with 150 μL of GC buffer containing (NH_4_)_2_SO_4_, NaH_2_PO_4_, and 2-ethylbutyric acid as an internal standard. SCFA concentrations were quantified using a headspace gas chromatography system equipped with a flame ionization detector (HSS-GC-FID; Agilent 7890B with 7697A headspace sampler, Agilent Technologies, USA). Separation was performed using an HP-INNOWax capillary column (30 m × 0.32 mm × 0.50 μm) with nitrogen as the carrier gas. The operating conditions were as follows: oven temperature, 85 °C; loop temperature, 90 °C; transfer-line temperature, 100 °C; detector temperature, 250 °C. The column temperature was initially set at 60 °C, increased to 140 °C at 30 °C/min, then to 170 °C at 30 °C/min, and finally to 180 °C at 40 °C/min and held for 0.75 min. SCFAs were identified and quantified using external standards, and concentrations were normalized to fecal weight and expressed as μmol/g feces.

### 2.8 Shotgun metagenomic sequencing

Fecal samples were collected from donor GK rats at week 2 following DLS, and from CTL and FMT recipient groups at baseline (week −2) and at the end of the experimental period (week 7) for shotgun metagenomic analysis. All samples were immediately frozen at −80 °C until further processing. Genomic DNA was extracted using the FastDNA™ SPIN Kit for Soil (MP Biomedicals, Santa Ana, CA, USA) according to the manufacturer’s instructions. DNA concentration was quantified using a Qubit 2.0 fluorometer (Invitrogen, Carlsbad, CA, USA). Sequencing libraries were prepared using the NEBNext Ultra II FS DNA Library Prep Kit for Illumina (New England Biolabs, MA, USA), following the manufacturer’s protocol optimized for ≤100 ng of input DNA. Paired-end sequencing (2 × 150 bp) was performed on the Illumina NovaSeq platform using a 300-cycle reagent kit. High-throughput sequencing generated an average of 42.4 million raw reads per sample across the pooled donor and recipient set (total n = 33; including 5 donor samples and 28 recipient samples) (range, 27.5–71.7 million reads). Initial preprocessing and host-read prefiltering of shotgun metagenomic sequencing data were performed using the nf-core/vshotflow pipeline (v1.0.0dev) implemented in Nextflow (v25.10.2) to ensure reproducibility. The preprocessing workflow included read quality control with fastp (v0.24.0), adapter and low-quality base trimming with Cutadapt (v5.0), and host-read removal by Bowtie2 alignment (v2.5.4) to the reference host genome. Only host-unmapped reads were retained for downstream bacteriome and virome assignment. Quality-control metrics were aggregated using MultiQC, and software versions were tracked to ensure traceability.

### 2.9 Construction of the custom viral reference database

A custom viral reference database was compiled from the National Center for Biotechnology Information (NCBI) Virus database (https://www.ncbi.nlm.nih.gov/labs/virus/), with the final data retrieval performed on April 26, 2025. The database was not restricted to eukaryotic viruses and included bacteriophage sequences as well as other viral sequences available in the NCBI Virus database. Sequences associated with SARS-CoV-2 (COVID-19) were excluded to prevent overrepresentation of this taxon, which would otherwise dominate the collection and bias downstream analyses.

The retrieved sequences were deduplicated to remove identical or redundant entries, retaining only unique representative sequences. To ensure sufficient sequence length for reliable downstream analysis, sequences shorter than 100 bp were filtered out. The resulting curated custom viral reference database, comprising non-redundant viral sequences of ≥100 bp, was used for viral read classification.

### 2.10 Microbiome and virome analysis

Taxonomic profiling of bacterial communities was performed using MetaPhlAn (v4.1.0), which utilizes clade-specific marker genes for species-level resolution. Viral sequences were identified from quality-filtered, host-unmapped reads using MEGABLAST (BLAST v2.15.0) against the custom viral reference database, with stringent thresholds of ≥80% query coverage and ≥90% sequence identity. Viral-classified reads were summarized at the sample level and normalized to relative abundance for downstream virome compositional analyses. Alignments that did not meet both query coverage and sequence identity thresholds were excluded from downstream analyses. Because viral-classified reads accounted for only a small fraction of total sequencing output, assembly-based viral contig reconstruction and validation using tools such as geNomad, VirSorter2, VIBRANT, or CheckV were not performed. Consequently, assembly metrics including contig N50 and chimeric contig assessment were not applicable.

All microbiome and virome analyses were conducted using R software (version 4.5.1; R Foundation for Statistical Computing, Vienna, Austria). α-diversity was assessed using Shannon and Simpson indices for both bacteriome and virome profiles at Pre-Tx and Post-Tx. Indices were calculated from relative abundance profiles, and rarefaction was not applied because the analyses were based on relative abundance rather than raw count tables. β-diversity was calculated using Bray–Curtis dissimilarity and visualized by principal coordinates analysis (PCoA). Statistical significance of group differences was assessed using PERMANOVA (permutational multivariate analysis of variance) with 999 permutations implemented via the adonis2 function in the vegan R package. To corroborate the PERMANOVA results, ANOSIM (analysis of similarities) was additionally performed using the same Bray–Curtis dissimilarity matrices with 999 permutations. To reduce noise from low-abundance taxa, filtering criteria were applied prior to analysis. For bacteriome analyses, taxa with a mean relative abundance ≥ 1% across all samples and present in > 50% of samples in at least one group were retained. For virome analyses, taxa with a mean relative abundance ≥ 0.1% and present in > 50% of samples per group were included. Differential abundance analysis was performed using Multivariable Association with Linear Models 2 (MaAsLin2). To account for the compositional nature of microbiome data, normalization was performed using the centered log-ratio (CLR) transformation, and no additional transformation step was applied. The recipient treatment group was included as a fixed effect, and no random effects were specified.

Taxa were ranked based on log_2_ fold change and Spearman correlation coefficients for visualization in S-plots. Nominal p-values and Benjamini–Hochberg false discovery rate (FDR)-adjusted q-values were reported for differential abundance and host–microbe correlation analyses. Results with nominal p< 0.05 but q ≥ 0.05 were interpreted as exploratory and were not considered statistically significant after multiple-testing correction. Statistical significance after FDR correction was defined as q< 0.05. Associations between bacterial taxa and host metabolic parameters (including glucose AUC_0–120_ and GLP-1 AUC_0–120_) were assessed using Spearman correlation analysis on CLR-transformed abundance data. All correlation analyses were conducted as two-tailed tests, and p-values were adjusted using the Benjamini–Hochberg method. Correlations with nominal p< 0.05 but q ≥ 0.05 were reported as exploratory associations, whereas q< 0.05 was considered statistically significant after FDR correction. To minimize false-positive associations, a more conservative threshold of q< 0.01 was specifically applied for the construction of bacteriome–virome co-occurrence networks.

### 2.11 Statistical analysis

All statistical analyses for host metabolic and histological parameters were performed using GraphPad Prism software (version 8.0; GraphPad Software, San Diego, CA, USA). Data are presented as mean ± standard deviation (SD). Longitudinal recipient metabolic outcomes were analyzed using two-way repeated-measures ANOVA. For AUC-derived outcomes and HOMA-β, group and period were used as factors, followed by Sidak’s multiple comparisons test where appropriate. OGTT glucose, insulin, and GLP-1 time-course curves were analyzed using two-way repeated-measures ANOVA with period and OGTT sampling time as repeated factors within each group, followed by Sidak’s multiple comparisons test. General longitudinal metabolic parameters, including body weight, food intake, fasting glucose, and non-fasting glucose, were analyzed using two-way repeated-measures ANOVA with group and week as factors. Donor OGTT glucose curves were analyzed using two-way repeated-measures ANOVA with donor time point and OGTT sampling time as repeated factors, followed by Dunnett’s multiple comparisons test versus baseline. Donor glucose AUC0–120 was analyzed using one-way repeated-measures ANOVA with Geisser–Greenhouse correction, followed by Dunnett’s multiple comparisons test versus baseline. Donor SCFA levels were compared between baseline and week 3 post-DLS using paired two-tailed t-tests for each SCFA species. Endpoint histological comparisons between CTL and FMT groups, including pancreatic insulin-positive area, colonic GLP-1–positive cells, duodenal morphometric parameters, and colonic mucin-positive area, were performed using Welch’s unpaired two-tailed t-test. For microbiome and virome relative-abundance data, group-wise comparisons were performed with non-parametric tests because the distributions were non-normal and the data were sparse. Specifically, between-group comparisons at each time point were conducted using the Mann–Whitney U test, and within-group longitudinal comparisons were performed using the Wilcoxon signed-rank test. All statistical tests were two-tailed, and a *p*-value< 0.05 was considered statistically significant.

## 3 Results

### 3.1 Experimental design and metabolic characterization of donor GK rats by DLS

**Figure 1:**
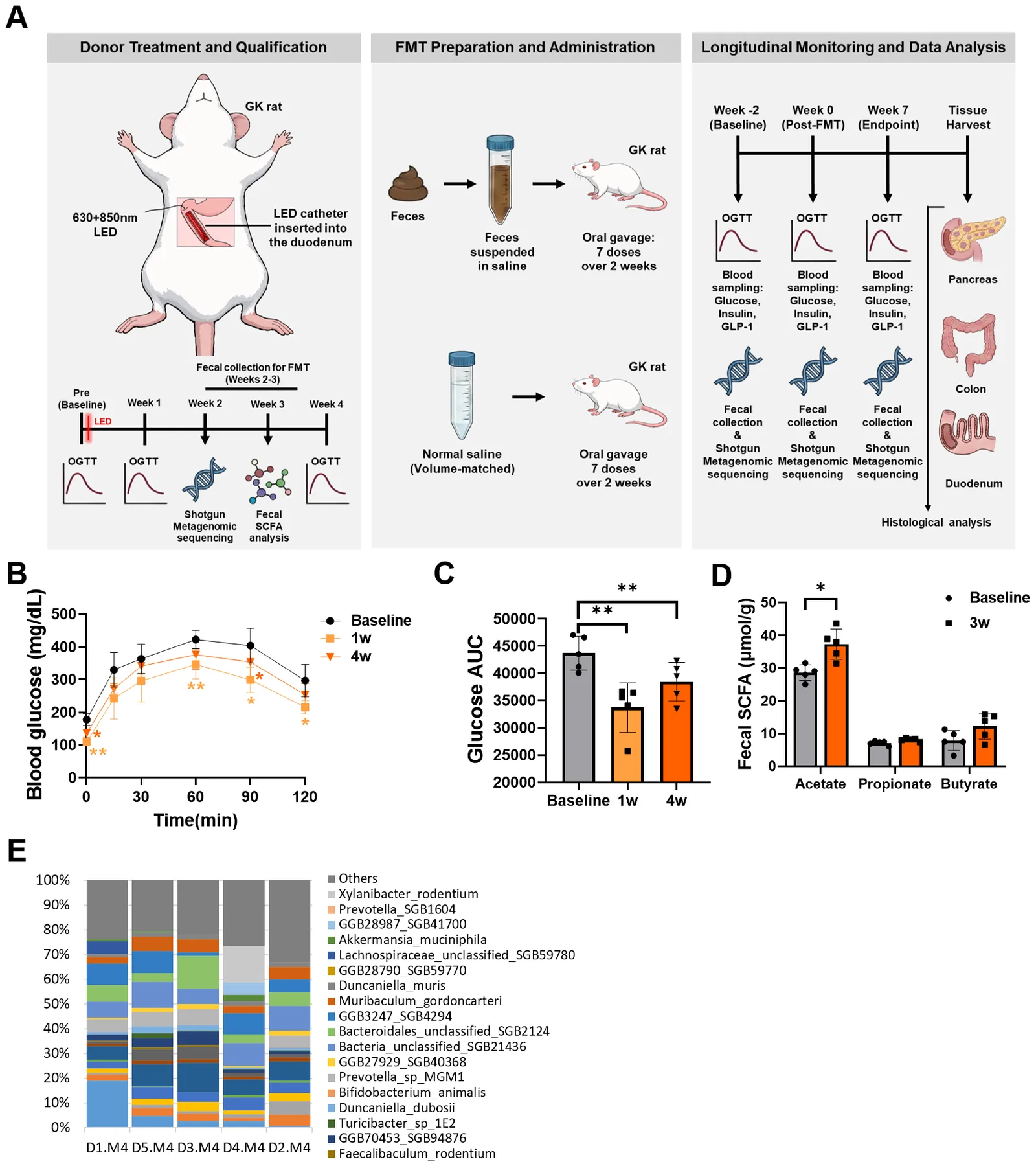
Experimental design and donor characterization following duodenal light stimulation (DLS). **(A)** Schematic overview of the experimental workflow. Donor GK rats were subjected to dual-wavelength DLS (630 and 850 nm), and donor metabolic responses were assessed by oral glucose tolerance test (OGTT). Donor feces collected at weeks 2–3 post-DLS were used for fecal microbiota transplantation (FMT) preparation; week 2 samples were used for shotgun metagenomic sequencing, and baseline/week 3 samples were used for short-chain fatty acid (SCFA) analysis. Recipient GK rats received pooled donor fecal suspension (FMT) or volume-matched saline (CTL) by oral gavage for 2 weeks and were assessed at week −2, week 0, and week 7. **(B)** Blood glucose time-course during OGTT in donor GK rats at baseline, week 1, and week 4 after DLS. **(C)** Glucose AUC0–120 during donor OGTT, expressed as mg/dL·min. **(D)** Fecal SCFA levels, including acetate, propionate, and butyrate, in donor GK rats at baseline and week 3 post-DLS, expressed as μmol/g feces. **(E)** Species-level fecal microbiome composition of donor GK rats at week 2 post-DLS, based on shotgun metagenomic sequencing. Data in **(B–D)** are presented as mean ± SD. Each dot represents an individual donor animal (n = 5). AUC values were calculated using the trapezoidal method. Donor OGTT glucose curves in **(B)** were analyzed using two-way repeated-measures ANOVA with donor time point and OGTT sampling time as repeated factors, followed by Dunnett’s multiple comparisons test versus baseline. Glucose AUC0–120 in **(C)** was analyzed using one-way repeated-measures ANOVA with Geisser–Greenhouse correction, followed by Dunnett’s multiple comparisons test versus baseline. SCFA levels in **(D)** were compared between baseline and week 3 using paired t-tests for each SCFA species. *P< 0.05, **P< 0.01 versus baseline.

The overall experimental design is shown in **Figure 1A**. Donor GK rats were subjected to DLS, and fecal samples collected at weeks 2–3 post-treatment were used for FMT preparation. Donor fecal samples collected at week 2 post-DLS were used for shotgun metagenomic sequencing, whereas fecal samples collected before DLS and at week 3 post-DLS were used for SCFA analysis. DLS-treated donor rats exhibited reduced OGTT glucose excursion, with a significant donor time point effect in the OGTT glucose curve analysis and significant reductions in glucose AUC0–120 at weeks 1 and 4 compared with baseline (adjusted p = 0.006 and p = 0.004, respectively; **Figures 1B, C**). At the time of fecal collection, fecal acetate levels were significantly increased (*p* = 0.037), whereas propionate and butyrate showed increasing trends (**Figure 1D**). Shotgun metagenomic profiling characterized the species-level bacterial composition of donor fecal samples, including GGB1521 SGB2103, GGB3247 SGB4294, *Bacteroidales* unclassified_SGB2124, and *Lactobacillus intestinalis* (**Figure 1E**).

### 3.2 FMT is associated with improved OGTT glucose profiles and modest early GLP-1-related changes in recipient GK rats

**Figure 2:**
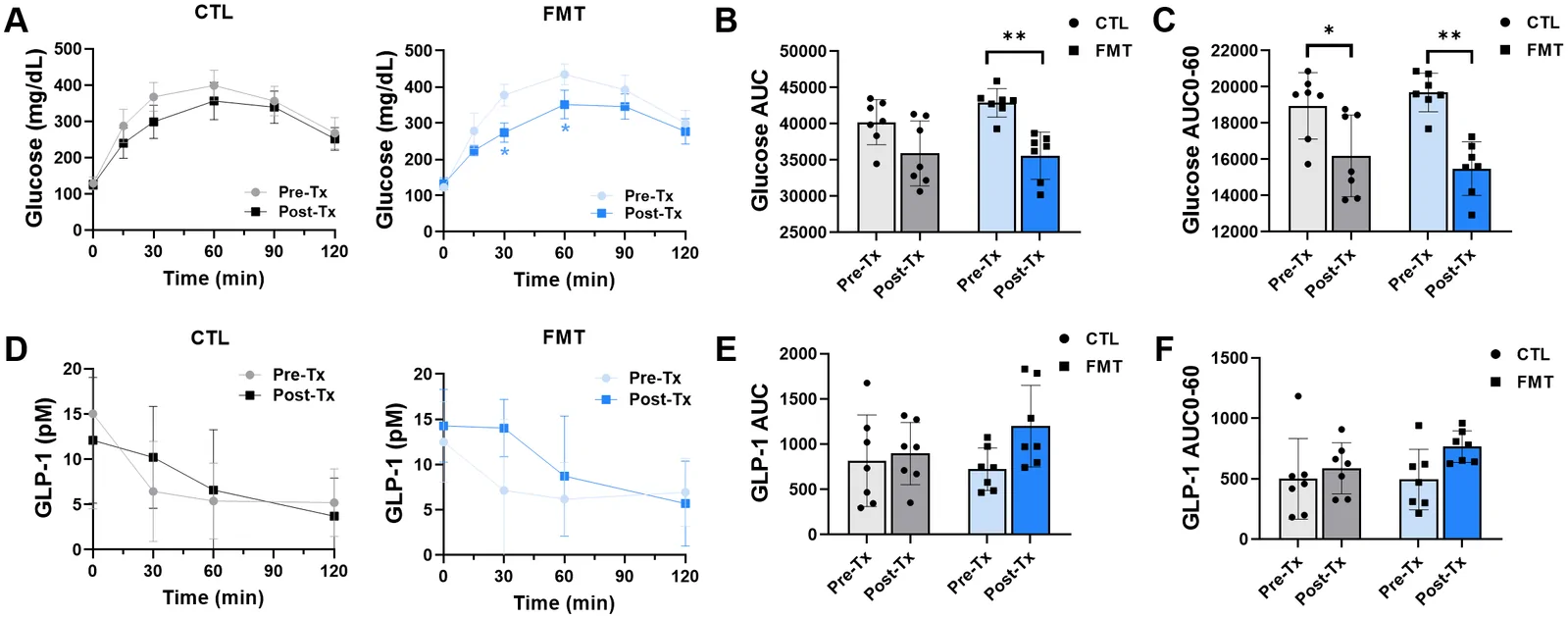
OGTT glucose profiles and GLP-1 responses in recipient GK rats after FMT. **(A)** Oral glucose tolerance test (OGTT) glucose curves in CTL and FMT recipient GK rats at Pre-Tx (week −2) and Post-Tx (week 7). **(B, C)** Total and early glucose exposure during OGTT, quantified as glucose AUC0–120 **(B)** and AUC0–60 **(C)**. **(D)** Plasma total GLP-1 concentration curves during OGTT in CTL and FMT recipient GK rats at Pre-Tx and Post-Tx. **(E, F)** Total and early GLP-1 exposure during OGTT, quantified as GLP-1 AUC0–120 **(E)** and AUC0–60 **(F)**. Blood glucose and plasma GLP-1 concentrations are expressed as mg/dL and pM, respectively. Glucose and GLP-1 AUC values are expressed as mg/dL·min and pM·min, respectively. AUC values were calculated using the trapezoidal method. Glucose AUC0–120 was calculated from values at 0, 30, 60, 90, and 120 min, and GLP-1 AUC0–120 was calculated from available values at 0, 30, 60, and 120 min. Early AUC0–60 values were calculated from values at 0, 30, and 60 min. Data are presented as mean ± SD. Each dot represents an individual animal (n = 7 per group). Time-course curves in **(A, D)** were analyzed within each group using two-way repeated-measures ANOVA with period and OGTT sampling time as repeated factors, followed by Sidak’s multiple comparisons test. AUC-derived outcomes in **(B, C, E, F)** were analyzed using two-way repeated-measures ANOVA with group and period as factors, followed by Sidak’s multiple comparisons test where appropriate. *P< 0.05, **P< 0.01 versus the corresponding Pre-Tx value within the same group.

General metabolic parameters, including body weight, food intake, fasting glucose, and non-fasting glucose levels, showed no significant group effects or group × week interactions throughout the experimental period (**Supplementary Figure 2**). Despite comparable general metabolic trajectories, FMT-recipient rats showed improved OGTT glucose profiles at Post-Tx. In the OGTT glucose time-course analysis, the FMT group showed a significant period effect and a significant period × OGTT sampling time interaction, with significant reductions at 30 and 60 min after glucose loading at Post-Tx compared with Pre-Tx (**Figure 2A**). For total glucose exposure, two-way repeated-measures ANOVA showed a significant period effect for glucose AUC0–120, whereas the group effect and group × period interaction were not significant. Sidak’s *post hoc* test showed that glucose AUC0–120 was significantly reduced in the FMT group (adjusted p = 0.002), whereas the reduction in the CTL group did not reach statistical significance (adjusted p = 0.060) (**Figure 2B**). The Pre-Tx glucose AUC0–120 difference between CTL and FMT groups was not statistically significant (adjusted p = 0.237). Early glucose exposure, assessed as glucose AUC0–60, also showed a significant period effect, with significant reductions in both CTL and FMT groups (adjusted p = 0.023 and p = 0.001, respectively), but without a significant group × period interaction (**Figure 2C**). For GLP-1 responses, total GLP-1 AUC0–120 did not show significant group, period, or group × period effects (**Figures 2D, E**). Early GLP-1 AUC0–60 showed a significant period effect and a non-significant trend toward an increase in the FMT group (adjusted p = 0.071), whereas the group × period interaction was not significant (**Figure 2F**). Together, these findings indicate that FMT recipients exhibited significant within-group improvement in OGTT glucose profiles, with a modest early GLP-1-related signal. However, because group × period interactions were not significant, these longitudinal metabolic changes should be interpreted as FMT-associated within-group changes rather than definitive between-group treatment effects.

### 3.3 FMT is associated with increased colonic GLP-1–positive cells

**Figure 3:**
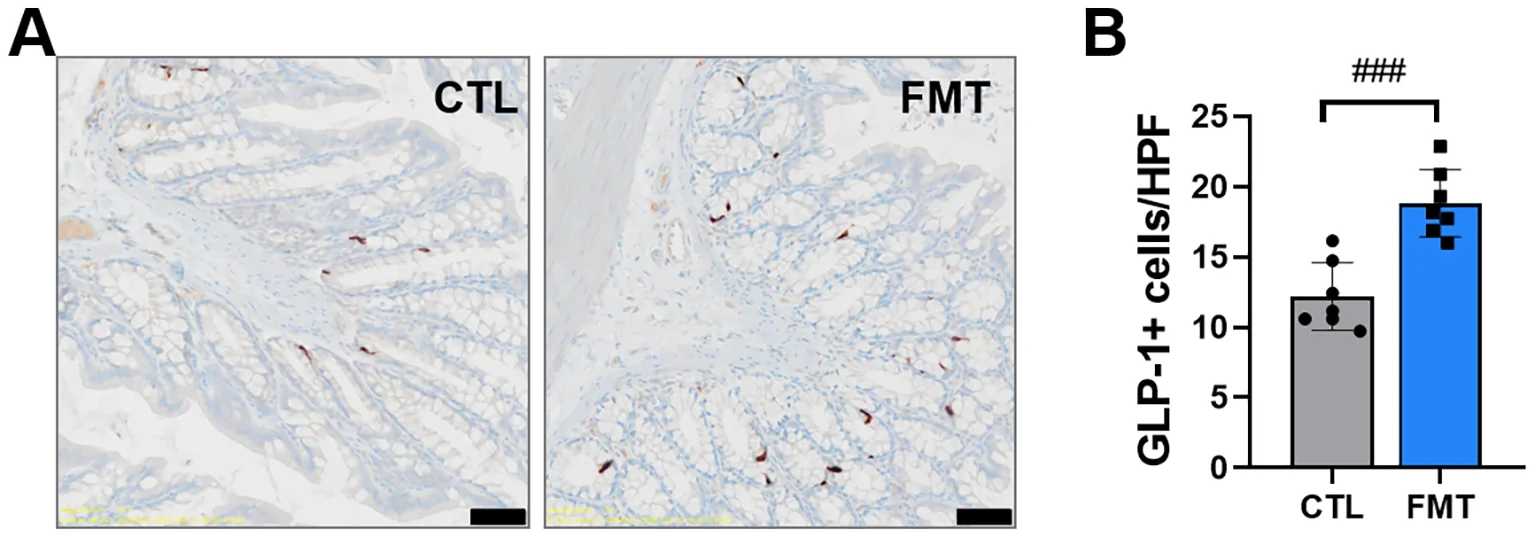
FMT is associated with increased colonic GLP-1–positive cells in recipient GK rats. **(A)** Representative immunohistochemical staining of GLP-1–positive cells in colonic sections from CTL and FMT groups at Post-Tx (week 7). Scale bar, 50 μm. **(B)** Quantification of GLP-1–positive cells per high-power field (HPF). Images were acquired using a VS200 slide scanner, and GLP-1–positive cells were manually counted in five non-overlapping HPFs per animal. Data are presented as mean ± SD. Each dot represents an individual animal (n = 7 per group). Statistical comparison between CTL and FMT groups was performed using Welch’s unpaired two-tailed t-test. ###P< 0.001 versus CTL.

Colonic GLP-1–producing enteroendocrine cells were quantified by immunohistochemistry. FMT-recipient rats had significantly more GLP-1–positive cells than CTL rats at the study endpoint (18.82 ± 2.50 *vs.* 12.18 ± 2.20 cells/HPF, +54.5%, *p*< 0.001) (**Figures 3A, B**). These findings indicate that FMT is associated with an increased number of colonic GLP-1–positive enteroendocrine cells in recipient GK rats.

### 3.4 Pancreatic insulin-positive β-cell area and insulin-related metabolic indices in recipient GK rats

**Figure 4:**
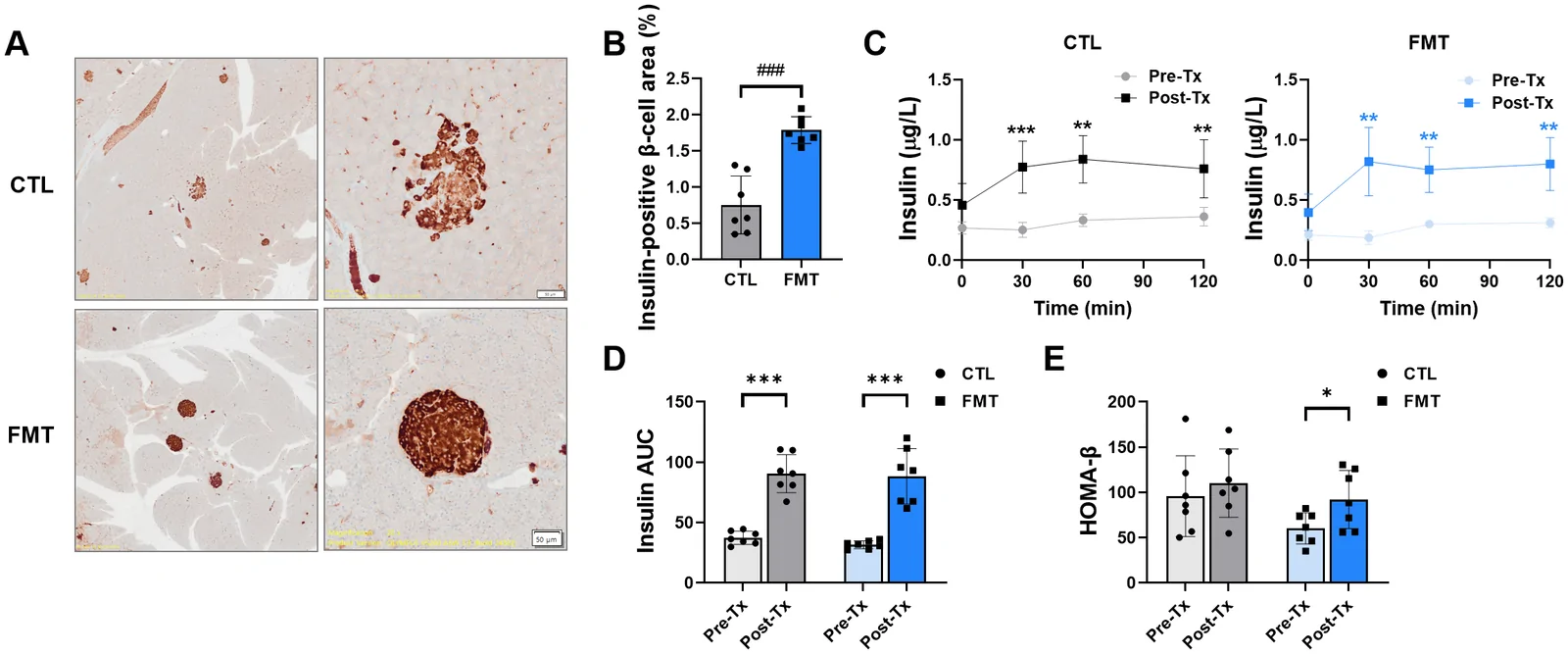
Pancreatic insulin-positive β-cell area and longitudinal insulin-related metabolic indices in recipient GK rats. **(A)** Representative insulin immunohistochemistry (IHC) images of pancreatic sections from CTL and FMT groups at Post-Tx (week 7). Scale bar, 50 μm. **(B)** Quantification of insulin-positive β-cell area, expressed as a percentage of total pancreatic tissue area. **(C)** Plasma insulin concentration curves during OGTT at Pre-Tx (week −2) and Post-Tx. Insulin concentrations are expressed as μg/L. **(D)** Total insulin exposure during OGTT, quantified as insulin AUC0–120 and expressed as μg/L·min. **(E)** Estimated β-cell function assessed by HOMA-β. Insulin AUC0–120 was calculated using the trapezoidal method based on available sampling points at 0, 30, 60, and 120 min. HOMA-β was calculated from fasting glucose and insulin levels. Images were acquired using a VS200 slide scanner and analyzed using Adobe Photoshop for quantification of insulin-positive β-cell area. Data are presented as mean ± SD. Each dot represents an individual animal (n = 7 per group). Endpoint insulin-positive β-cell area in **(B)** was compared using Welch’s unpaired two-tailed t-test. Insulin curves in **(C)** were analyzed within each group using two-way repeated-measures ANOVA with period and OGTT sampling time as repeated factors, followed by Sidak’s multiple comparisons test. Insulin AUC0–120 and HOMA-β in **(D, E)** were analyzed using two-way repeated-measures ANOVA with group and period as factors, followed by Sidak’s multiple comparisons test where appropriate. *P< 0.05, **P< 0.01, ***P< 0.001 versus the corresponding Pre-Tx value within the same group; ###P< 0.001 versus CTL.

Pancreatic histology revealed a significantly greater insulin-positive β-cell area in FMT-recipient rats than in CTL rats at the study endpoint (1.79 ± 0.19% *vs.* 0.75 ± 0.36%, +139.0%, *p*< 0.001) (**Figures 4A, B**). Plasma insulin responses during OGTT increased from Pre-Tx to Post-Tx in both CTL and FMT groups (**Figure 4C**). Total insulin AUC0–120 showed a significant period effect, whereas the group effect and group × period interaction were not significant. Sidak’s *post hoc* test showed significant increases in insulin AUC0–120 in both CTL and FMT groups (**Figure 4D**). HOMA-β also showed a significant period effect and increased significantly within the FMT group (adjusted p = 0.010), whereas no significant change was observed in the CTL group (adjusted p = 0.272) (**Figure 4E**). However, the group × period interaction for HOMA-β was not significant. These findings indicate that FMT is associated with increased pancreatic insulin-positive β-cell area and a within-group increase in estimated β-cell function, while insulin exposure during OGTT increased over time in both groups and was not specific to FMT. Because the group × period interaction was not significant for HOMA-β, the within-group increase observed in the FMT group does not constitute evidence that the longitudinal HOMA-β change was significantly different between groups.

### 3.5 FMT is associated with an increased duodenal villus-to-crypt ratio and a numerical increase in colonic mucin-positive area

**Figure 5:**
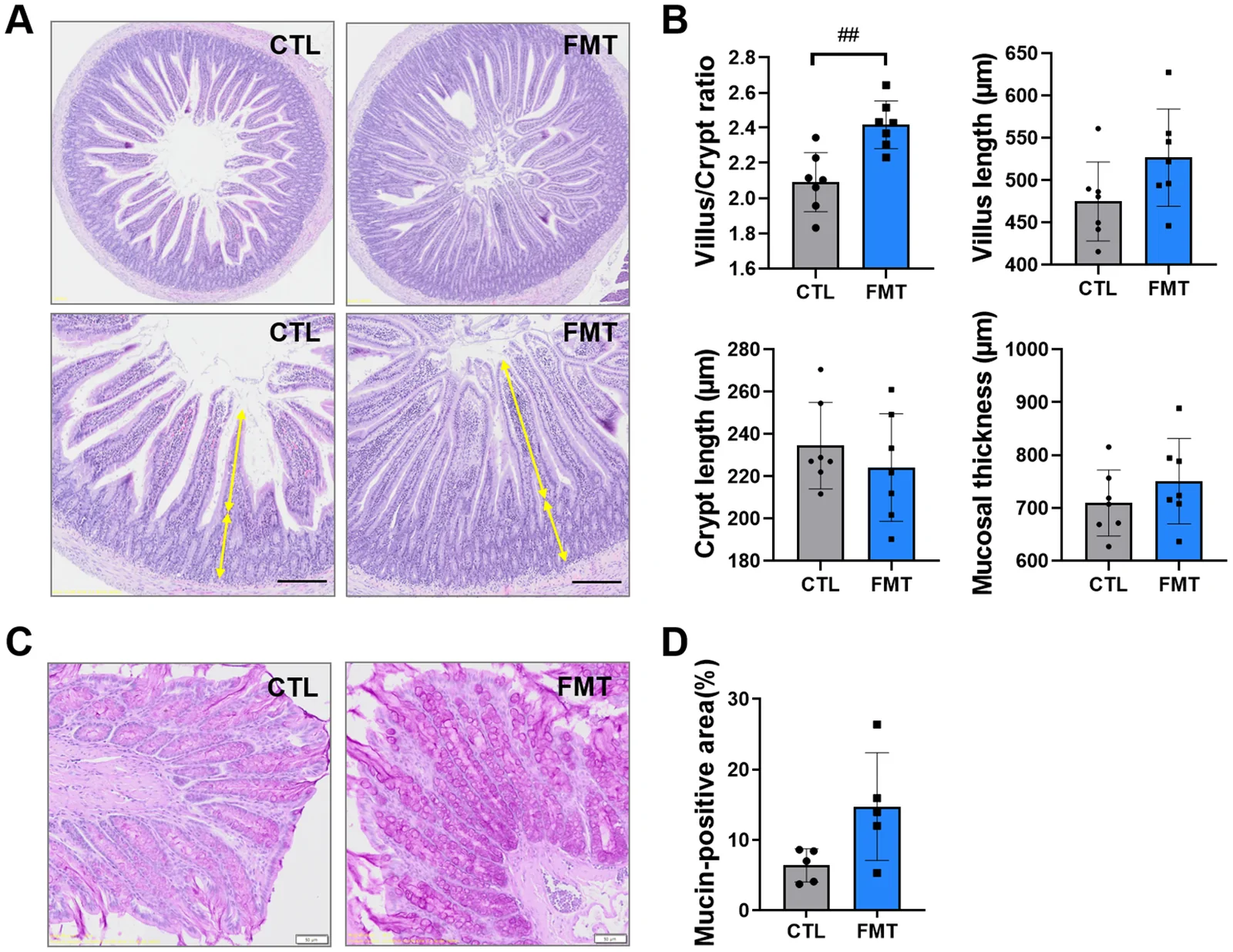
FMT is associated with duodenal architectural changes and colonic mucin staining in recipient GK rats. **(A)** Representative hematoxylin and eosin (H&E)–stained duodenal sections from CTL and FMT groups at Post-Tx (week 7), shown at low and high magnification. Yellow arrows indicate representative villus height. Scale bars are indicated in the images. **(B)** Quantitative analysis of duodenal morphology, including villus-to-crypt ratio, villus length, crypt length, and mucosal thickness. **(C)** Representative periodic acid–Schiff (PAS)–stained colonic sections from CTL and FMT groups at Post-Tx. Scale bar, 50 μm. **(D)** Quantification of mucin-positive area in colonic sections, expressed as a percentage of high-power field (HPF) area. HPF corresponds to 487 × 487 μm. Images were acquired using a VS200 slide scanner and analyzed using HALO for duodenal morphology and ImageJ for mucin-positive area. Data are presented as mean ± SD. Each dot represents an individual animal. Duodenal morphology was analyzed in n = 7 animals per group, and colonic mucin-positive area was analyzed in n = 5 animals per group. Statistical comparisons between CTL and FMT groups at Post-Tx were performed using Welch’s unpaired two-tailed t-test. ##P< 0.01 versus CTL.

Histological analysis of the duodenum demonstrated structural differences between CTL and FMT groups at the study endpoint. Villus height was higher in FMT-recipient rats than in CTL rats, although this difference did not reach statistical significance (FMT: 526.50 ± 57.48 *vs.* CTL: 474.83 ± 46.60 μm; Welch’s *t*-test, *p* = 0.091). The villus-to-crypt ratio was significantly increased in the FMT group (FMT: 2.42 ± 0.14 *vs.* CTL: 2.09 ± 0.17; *p* = 0.002), indicating altered duodenal epithelial architecture (**Figures 5A, B**). Crypt depth (FMT: 224.10 ± 25.40 *vs.* CTL: 234.45 ± 20.49 μm; *p* = 0.418) and mucosal thickness (FMT: 750.60 ± 80.75 *vs.* CTL: 709.29 ± 62.45 μm; *p* = 0.307) were comparable between groups, suggesting that the increased villus-to-crypt ratio reflected higher villus height rather than changes in crypt depth. In the colon, PAS staining showed a higher mucin-positive area in the FMT group, although this difference did not reach statistical significance (*p* = 0.070) (**Figures 5C, D**). Collectively, these data indicate that FMT is associated with an increased duodenal villus-to-crypt ratio and a trend toward increased colonic mucin-positive area in recipient GK rats.

### 3.6 FMT is associated with fecal bacteriome differences and exploratory host–microbe associations in recipient GK rats

**Figure 6:**
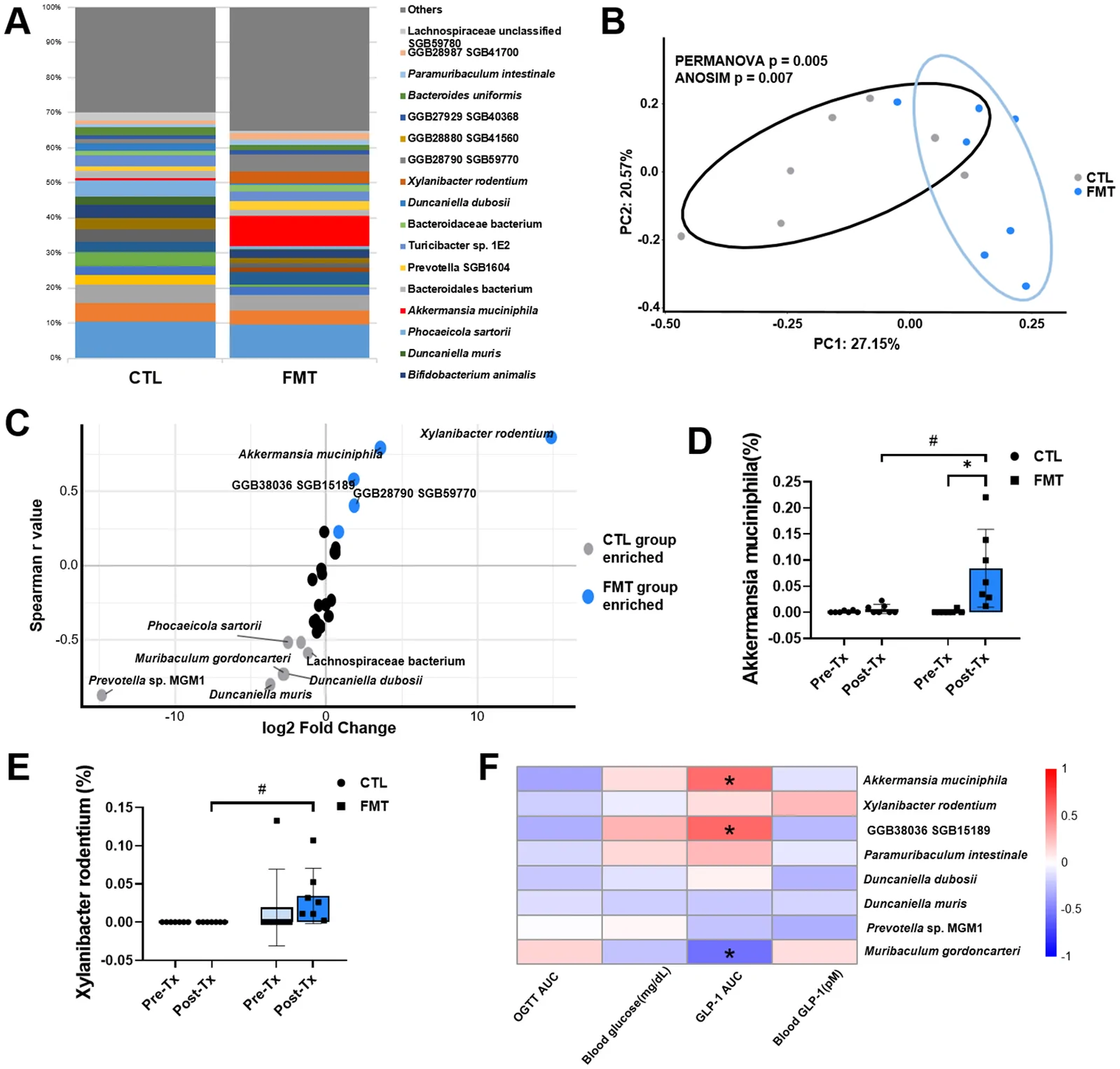
FMT is associated with fecal bacteriome differences and exploratory host–microbe associations in recipient GK rats. **(A)** Relative abundance of major bacterial taxa at the species level in CTL and FMT fecal samples at Post-Tx, based on shotgun metagenomic analysis. **(B)** Principal coordinates analysis (PCoA) of species-level bacteriome β-diversity based on Bray–Curtis dissimilarity. Group separation was assessed using PERMANOVA and corroborated using ANOSIM. **(C)** Bacterial taxa showing nominal group-associated differences based on log_2_ fold change and correlation coefficients (S-plot). **(D, E)** Relative abundance of *Akkermansia muciniphila* **(D)** and *Xylanibacter rodentium* **(E)** at Pre-Tx and Post-Tx in CTL and FMT groups. **(F)** Spearman correlation heatmap showing exploratory associations between selected bacterial taxa and host metabolic parameters, including glucose AUC0–120 and GLP-1 AUC0–120. Data in **(D, E)** are presented as individual values. Between-group comparisons at each time point were performed using the Mann–Whitney U test, and within-group comparisons were analyzed using the Wilcoxon signed-rank test. Colors in **(F)** indicate Spearman correlation coefficients, and asterisks indicate nominal significance before FDR correction (*P< 0.05, **P< 0.01). Pre-Tx corresponds to week −2 before FMT, and Post-Tx corresponds to week 7.

Shotgun metagenomic analysis at the species level revealed differences in fecal bacteriome composition between CTL and FMT groups (**Figure 6A**). Bacteriome α-diversity, assessed using Shannon and Simpson indices, did not differ significantly across groups or time points (**Supplementary Figures 4A, B**). PCoA based on Bray–Curtis dissimilarity demonstrated a significant separation between the two groups by PERMANOVA (R² = 0.271, p = 0.005), and this separation was further supported by ANOSIM (R = 0.349, p = 0.007), supporting group-associated differences in bacteriome community structure (**Figure 6B**). Differential abundance analysis using the MaAsLin2 model identified several taxa with nominal group-associated differences between CTL and FMT groups (**Figure 6C**; **Table 1**). After Benjamini–Hochberg FDR correction, only *Prevotella* sp. MGM1 remained statistically significant (p = 0.001, q = 0.029), whereas the other taxa, including *Akkermansia muciniphila* and *Xylanibacter rodentium*, did not remain significant based on q-values. Longitudinal analysis further showed that these compositional differences were primarily observed at Post-Tx (week 7), with minimal differences at baseline (week −2). Direct group-wise comparisons showed nominally higher *A. muciniphila* abundance in the FMT group at Post-Tx than in CTL (p = 0.032), accompanied by a nominal within-group increase from baseline in the FMT group (p = 0.025) (**Figure 6D**). Similarly, *X. rodentium* was undetectable in the CTL group but was detected in multiple FMT recipients at Post-Tx, showing a nominal between-group difference (p = 0.046) (**Figure 6E**). These patterns are consistent with FMT-associated species-level enrichment or expansion. However, because strain-level donor–recipient tracking was not performed, they cannot establish definitive donor-derived engraftment. Correlation analysis revealed nominal microbiome–host associations. *A. muciniphila* showed a nominal positive association with GLP-1 AUC (r = 0.574, nominal p = 0.032), but this association did not survive FDR correction (q = 0.436). Similarly, nominal associations between GLP-1 AUC and other selected taxa did not remain significant after FDR correction (**Figure 6F**). A descriptive species-level comparison between donor fecal samples and FMT recipient samples at Post-Tx is provided in **Supplementary Figure 5**. Together, these findings indicate that FMT was associated with fecal bacteriome community-level differences and post-FMT species-level enrichment patterns, including one FDR-significant taxon and several nominally associated taxa.

**Table 1:** Bacterial and viral taxa showing nominal group-associated differences between CTL and FMT groups identified by MaAsLin2.

Taxon	Estimate	SE	*p*-value	*q*-value	CTL (n=7)		FMT (n=7)	
					Mean	SD	Mean	SD
Bacteria								
*Akkermansia muciniphila*	0.039	0.013	0.012	0.118	0.625	0.876	8.439	7.448
*Xylanibacter rodentium*	0.019	0.007	0.020	0.132	0.000	0.000	3.434	3.616
GGB38036 SGB15189	0.011	0.004	0.023	0.132	0.593	0.676	2.384	1.975
*Paramuribaculum intestinale*	0.004	0.002	0.042	0.151	0.839	0.556	1.398	0.764
*Duncaniella dubosii*	−0.007	0.003	0.034	0.143	2.097	1.449	0.349	0.517
*Duncaniella muris*	−0.009	0.003	0.008	0.113	2.180	1.492	0.192	0.183
*Prevotella* sp. MGM1	−0.012	0.003	0.001	0.029	2.583	1.173	0.000	0.000
*Muribaculum gordoncarteri*	−0.015	0.006	0.035	0.143	3.997	3.637	0.612	0.392
Viruses								
Akkermansia phage	0.004	0.002	0.042	0.434	0.039	0.051	0.719	0.805
Moraxella phage	−3.62E−04	1.38E−04	0.022	0.434	0.196	0.055	0.124	0.016

Relative abundance is presented as percentage (%). Values are shown as mean ± SD for each group (CTL, n = 7; FMT, n = 7). Estimate represents the effect size from the MaAsLin2 model, and SE represents the standard error. Positive estimates indicate higher abundance in the FMT group, whereas negative estimates indicate higher abundance in the CTL group. Reported p-values are nominal p-values from the MaAsLin2 model, and q-values represent Benjamini–Hochberg FDR-adjusted p-values. Taxa with nominal p< 0.05 were included in the table, and q< 0.05 was considered significant after FDR correction. Estimate, effect size; SE, standard error; SD, standard deviation; FDR, false discovery rate.

### 3.7 Virome profiling identifies nominal viral taxon differences without global virome separation

**Figure 7:**
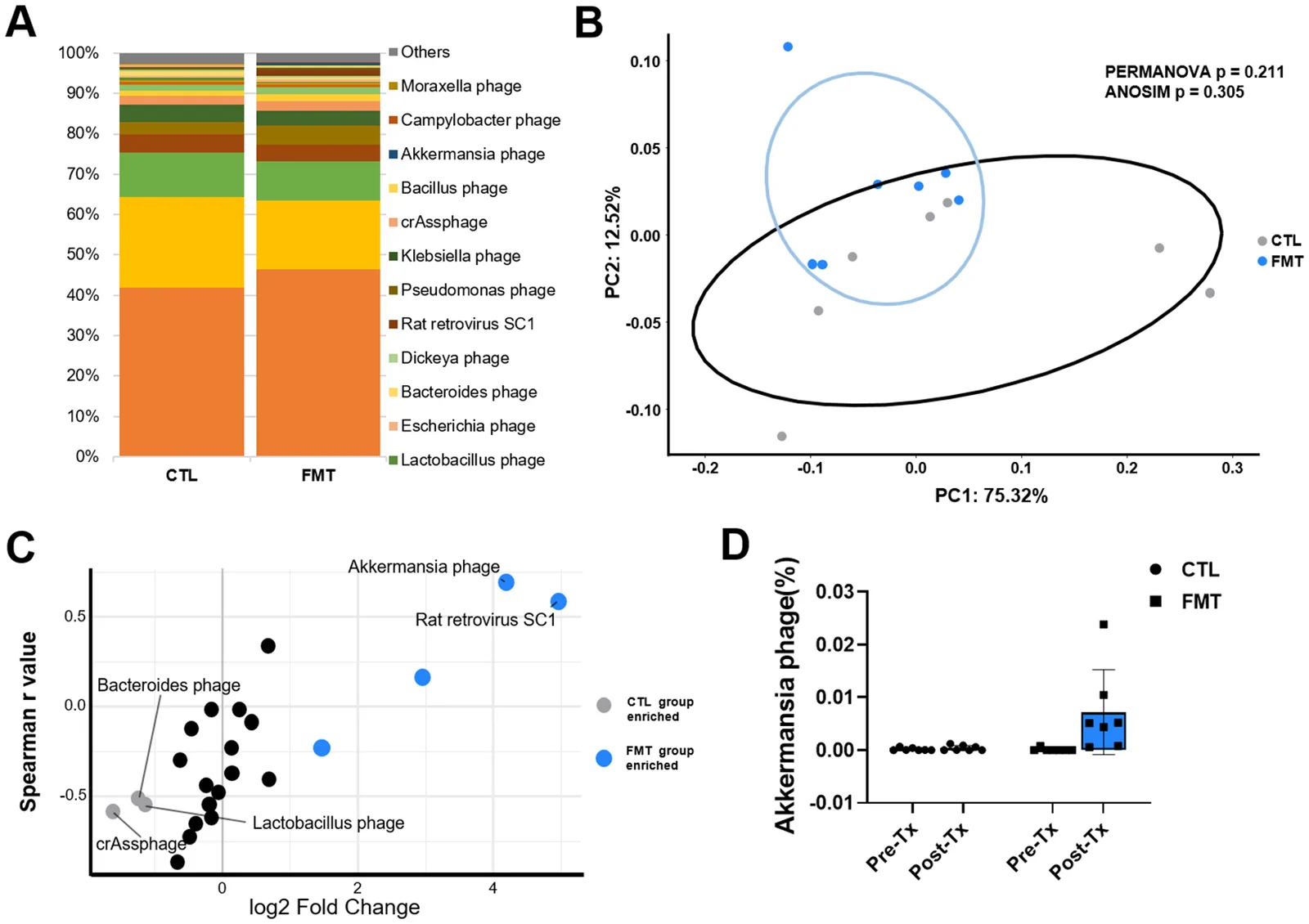
Virome profiling identifies nominal viral taxon differences without global virome separation in recipient GK rats. **(A)** Relative abundance of major viral taxa in CTL and FMT fecal samples at Post-Tx, based on read-level shotgun metagenomic viral profiling. **(B)** Principal coordinates analysis (PCoA) of viral β-diversity based on Bray–Curtis dissimilarity. Group separation was assessed using PERMANOVA and corroborated using ANOSIM. **(C)** Viral taxa showing nominal group-associated differences based on log_2_ fold change and correlation coefficients (S-plot). **(D)** Relative abundance of Akkermansia phage at Pre-Tx and Post-Tx in CTL and FMT groups. Data in **(D)** are presented as individual values. Between-group comparisons at each time point were performed using the Mann–Whitney U test, and within-group comparisons were analyzed using the Wilcoxon signed-rank test. Pre-Tx corresponds to week −2 before FMT, and Post-Tx corresponds to week 7.

Shotgun read-level virome profiling revealed that bacteriophages were the predominant viral taxa in fecal samples from both CTL and FMT groups (**Figure 7A**). The mean number of viral-classified reads was 96,331 reads per sample, with a range of 38,090–182,338 reads per sample, corresponding to approximately 0.23% of the average total raw read depth. Virome α-diversity, assessed using Shannon and Simpson indices, did not differ significantly across groups or time points (**Supplementary Figures 4C, D**). PCoA based on Bray–Curtis dissimilarity showed no significant separation between CTL and FMT groups by PERMANOVA (R² = 0.124, p = 0.211), and this result was consistent with ANOSIM (R = 0.037, p = 0.305) (**Figure 7B**). Differential abundance analysis using the MaAsLin2 model identified viral taxa with nominal group-associated differences between CTL and FMT groups (**Figure 7C**; **Table 1**). *Akkermansia phage* showed a nominal positive association with FMT (p = 0.042, q = 0.434), whereas *Moraxella phage* showed a nominal negative association (p = 0.022, q = 0.434); however, neither viral taxon remained significant after FDR correction. Direct group-wise comparisons showed that *Akkermansia phage* abundance was higher in the FMT group at Post-Tx than in CTL, although this difference did not reach statistical significance (p = 0.060) (**Figure 7D**). *Moraxella phag*e showed a lower abundance in the FMT group in direct comparisons, consistent with the direction of the MaAsLin2 result. These results indicate that no broad FMT-associated shift in overall virome composition was detected, while selected viral taxa showed nominal model-based or direct group-wise differences that did not remain significant after FDR correction.

### 3.8 FMT is associated with altered exploratory bacteriome–virome association networks

**Figure 8:**
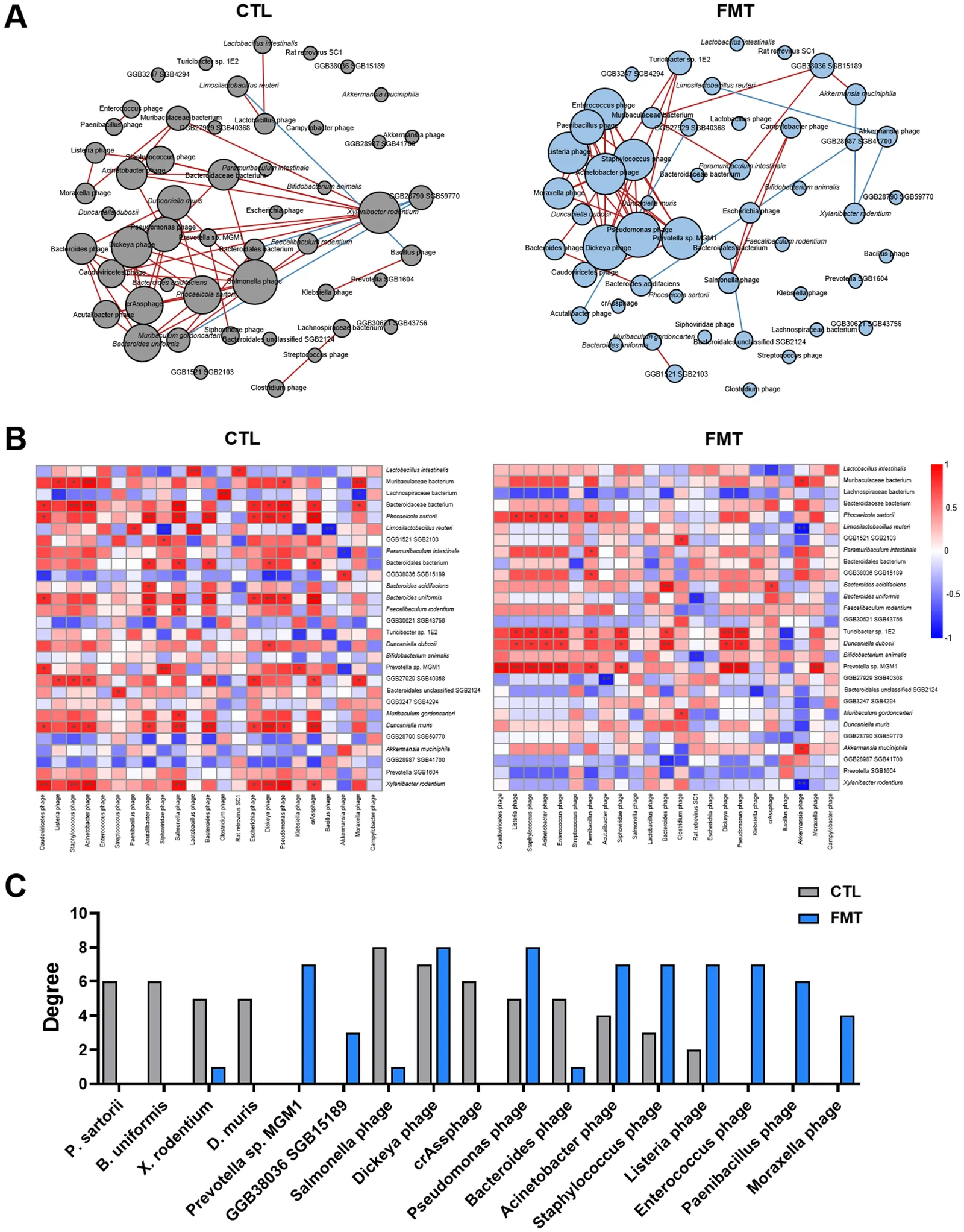
Exploratory bacteriome–virome association networks in recipient GK rats after FMT. **(A)** Spearman correlation-based association networks of fecal bacteriome and virome profiles in CTL and FMT groups at Post-Tx, based on shotgun metagenomic data. Networks were constructed using thresholds of |ρ| ≥ 0.40 and q< 0.01. Node size represents degree (number of significant associations), and edges indicate significant correlations (red, positive; blue, negative). **(B)** Heatmaps of bacteriome–virome correlations within each group. Colors represent Spearman correlation coefficients, and asterisks indicate FDR-adjusted significance (*q< 0.05, **q< 0.01). **(C)** Degree-based comparison of highly connected taxa in CTL and FMT association networks. Taxa shown represent nodes with the highest degree in each network. *B. uniformis*, *Bacteroides uniformis*; *P. sartorii*, *Phocaeicola sartorii*; *X. rodentium*, *Xylanibacter rodentium*; *D. muris*, *Duncaniella muris*.

To explore group-associated microbial association patterns, bacteriome–virome association networks were constructed using Spearman correlation-based co-occurrence analysis (|ρ| ≥ 0.40, q< 0.01) at Post-Tx (**Figure 8A**; **Supplementary Table 1**). Although the total number of significant associations was comparable between groups (CTL, 57 edges; FMT, 56 edges), the edge composition and node connectivity patterns differed between them. In the CTL group, the network showed a higher proportion of bacteria–virus associations, whereas the FMT group exhibited a higher proportion of virus–virus associations. Specifically, the proportion of virus–virus associations increased from 29.8% (17/57 edges) in CTL to 55.4% (31/56 edges) in FMT, while the average bacterial node degree decreased from 3.5 in CTL to 2.1 in FMT. Heatmap analysis further supported these observations by showing distinct bacteriome–virome correlation patterns between CTL and FMT groups (**Figure 8B**). Degree-based comparison of highly connected taxa demonstrated that taxa with the highest degree differed between groups, indicating group-specific differences in network connectivity (**Figure 8C**). These findings indicate that FMT was associated with altered exploratory bacteriome–virome association patterns, characterized by increased virus–virus connectivity and reduced bacterial node connectivity, rather than broad changes in overall virome composition.

## 4 Discussion

The gut microbiota is a dynamic, metabolically active ecosystem that plays an important role in regulating host metabolism, immunity, and intestinal epithelial integrity (Kayama et al., 2020; Gieryńska et al., 2022). Alterations in microbial community structure have been implicated in nutrient sensing, enteroendocrine hormone secretion, barrier function, and low-grade inflammation, processes that collectively shape glucose metabolism (Kayama et al., 2020; Qi et al., 2021).

Impaired GLP-1 secretion has been reported in patients with type 2 diabetes, potentially reflecting dysfunction of intestinal enteroendocrine L cells (Wang et al., 2023). Emerging evidence indicates that the gut microbiota contributes to enteroendocrine regulation, including GLP-1 secretion (Cani et al., 2013; Zeng et al., 2024). Microbial metabolites such as SCFAs stimulate L cells via G protein–coupled receptors, thereby enhancing GLP-1 secretion (Tolhurst et al., 2012; Puddu et al., 2014). The gut microbiota may also indirectly influence L-cell function by modulating the intestinal environment, including mucus dynamics and epithelial integrity, both of which are known to affect enteroendocrine signaling (Qi et al., 2021; Dmytriv et al., 2024).

In the present study, we found that FMT from DLS-conditioned donor GK rats was associated with improved glucose tolerance and incretin-related intestinal changes in diabetic recipient GK rats. These effects occurred without significant changes in fasting glucose levels, suggesting that the FMT-associated metabolic phenotype was more evident during glucose challenge than under fasting conditions. Glucose AUC0–120 was significantly reduced within the FMT group by *post hoc* testing, whereas the group × period interaction was not significant; the data therefore do not support a statistically significant longitudinal between-group treatment effect of FMT, and the improvement in glucose tolerance should be interpreted as a within-group longitudinal change. Consistent with the overall exploratory design of this study, these glucose-related findings are presented as exploratory. Although both CTL and FMT groups exhibited increased insulin AUC during OGTT over time, with no significant group × period interaction, this pattern may reflect longitudinal compensatory insulin secretion in GK rats rather than an FMT-specific insulinotropic effect. In parallel, HOMA-β increased within the FMT group, and pancreatic insulin-positive area was greater in FMT recipients at the study endpoint, suggesting improvement in estimated β-cell-related parameters without establishing enhanced β-cell functional responsiveness. Because the group × period interaction was not significant for either insulin AUC0–120 or HOMA-β, the within-group changes observed in the FMT group cannot be interpreted as evidence that FMT produced a significantly greater longitudinal effect than the control condition (Nieuwenhuis et al., 2011).

These metabolic changes were accompanied by an increased number of colonic GLP-1–positive cells and changes in intestinal architecture, which may support an intestinal environment favorable to enteroendocrine regulation (Gribble, 2012; Nie et al., 2023).

Donor animals showed increased fecal acetate levels, with upward trends in other major SCFAs, whereas recipient SCFA levels were not significantly altered at the study endpoint (**Supplementary Figure 3**). This discrepancy may reflect differences in sampling timing or transient post-FMT metabolic dynamics, whereby donor-associated metabolic features may not be maintained or detectable in recipient feces at the endpoint. SCFAs reduce intestinal pH and modulate epithelial function, including mucosal integrity and mucus production (Walker et al., 2005; Burger-van Paassen et al., 2009). Therefore, the donor SCFA profile provides a plausible metabolic context for FMT-associated intestinal changes, although direct evidence linking donor SCFAs to recipient intestinal remodeling was not obtained in the present study.

At the bacteriome level, FMT was associated with community-level compositional differences rather than broad changes in α-diversity or definitive donor-derived transfer. The absence of significant changes in bacteriome α-diversity suggests that these differences reflected between-sample compositional restructuring rather than broad changes in within-sample bacterial diversity. Among the taxa showing nominal group-associated differences, *A. muciniphila* showed higher abundance in FMT recipients at Post-Tx and increased from baseline within the FMT group; however, this difference did not remain significant after FDR correction in the differential abundance analysis. This finding is biologically plausible given the established roles of *A. muciniphila* in mucosal homeostasis and metabolic regulation (Derrien et al., 2011). Notably, *A. muciniphila* abundance showed a nominal positive association with GLP-1 AUC (r = 0.574, nominal p = 0.032), but this association did not survive FDR correction (q = 0.436), indicating that this host–microbe association should be interpreted as exploratory. A descriptive species-level comparison between donor fecal samples and FMT recipient samples showed partial overlap in detected bacterial taxa at Post-Tx. However, because this comparison was not strain-resolved, it cannot distinguish donor-derived acquisition from expansion of pre-existing low-abundance recipient populations and was not used as evidence of definitive donor-derived engraftment. These observations are compatible with host-dependent ecological filtering after FMT rather than simple uniform transfer of donor microbiota (Smillie et al., 2018; Porcari et al., 2023). In this context, the post-FMT increase of *A. muciniphila* may reflect species-level expansion within an altered mucosal environment rather than confirmed donor-derived engraftment (Derrien et al., 2011). Collectively, these findings support a hypothesis-generating framework in which donor-associated microbial metabolites and FMT-associated intestinal remodeling may create a mucosal environment favorable for changes in mucin-associated taxa such as *A. muciniphila*, which depends on mucin availability for growth (Geerlings et al., 2018; Van Herreweghen et al., 2018). Although direct measurements of luminal pH, longitudinal mucus dynamics, or strain-resolved donor–recipient tracking were not performed, this framework links the observed donor microbial features, recipient intestinal remodeling, and selective bacteriome restructuring after FMT while acknowledging that donor origin and causality remain unresolved. Importantly, the short-read shotgun metagenomic approach used in this study inherently lacks the resolution required for strain-level donor–recipient tracking. Tools such as StrainPhlAn or inStrain, which leverage single-nucleotide variant (SNV) profiling, or long-read sequencing platforms, would be necessary to resolve strain-level identity and confirm donor origin of specific taxa.

Accumulating experimental and translational evidence indicates that *A. muciniphila* is not only a marker of mucosal health but also an active modulator of host metabolism, with reported associations with improvements in glucose homeostasis and GLP-1 regulation (Everard et al., 2013; Depommier et al., 2019; Yoon et al., 2021). Notably, P9, a secreted protein derived from this species, has been shown to stimulate enteroendocrine L cells via receptor-mediated signaling, thereby enhancing GLP-1 secretion (Yoon et al., 2021). Taken together, these prior observations and the parallel changes in intestinal morphology, fecal bacteriome composition, colonic GLP-1–positive cells, and early GLP-1-related responses observed in the present study provide biological context for the nominal *A. muciniphila*–GLP-1 association observed here. However, because this association did not survive FDR correction, it should be interpreted as a hypothesis-generating signal rather than evidence of a direct mechanistic role for *A. muciniphila* in GLP-1 regulation. Accordingly, the extent of discussion devoted to *A. muciniphila* in this manuscript is intended to be proportionate to its statistical support; because this taxon did not survive FDR correction in either the differential abundance analysis (q = 0.118) or the host–microbe correlation analysis (q = 0.436), the findings are presented within a clearly labeled exploratory framework rather than as a central interpretive theme.

In contrast, no significant global shift in the detected fecal virome composition was observed after FMT. Consistent with this finding, virome α-diversity also did not differ significantly across groups or time points, supporting the interpretation that FMT was not associated with broad changes in fecal virome diversity. Model-based analysis identified only nominal differences in selected annotated viral taxa, and these differences did not remain significant after FDR correction. This pattern suggests that virome-level differences were limited in the present dataset. Moreover, because the virome analysis was performed using total shotgun metagenomic data and viral-classified reads accounted for only approximately 0.23% of total raw reads on average, the viral findings should be interpreted as shotgun read-level exploratory results rather than assembly-validated virome reconstruction. In addition, the alignment thresholds used for viral read classification (≥80% query coverage, ≥90% sequence identity), while intended to reduce false-positive assignments, are relatively permissive for viral taxonomy given the high sequence diversity and rapid evolutionary rates characteristic of bacteriophage genomes. Furthermore, the low viral read fraction may introduce detection bias toward phage taxa that are well represented in the reference database, while under-representing novel or divergent viral lineages. Because no *de novo* assembly was performed, chimeric contig assessment was not applicable.

Given the role of bacteriophages in modulating bacterial populations and maintaining community stability (Hsu et al., 2019; Sutton and Hill, 2019), these nominal viral taxon differences may reflect localized ecological shifts within the gut microbiome. Importantly, network-based analysis suggested altered bacteriome–virome association patterns despite minimal changes in virome composition. Although the total number of correlations was comparable between groups, the FMT group exhibited differences in correlation-based network structure, including increased virus–virus connectivity.

Notably, *Akkermansia*-associated bacteriophages showed a nominal model-based positive association with FMT in parallel with the nominal increase in *A. muciniphila*, although this viral association did not remain significant after FDR correction and was not statistically significant in direct group-wise comparisons. This co-occurrence is compatible with reported phage–host association patterns, in which expansion of specific bacterial taxa is accompanied by corresponding phage populations (Chevallereau et al., 2022). Alternatively, bacteriophages have been proposed to contribute to the regulation of microbial community structure through density-dependent control mechanisms, thereby influencing the persistence or turnover of specific taxa within the gut ecosystem (De Sordi et al., 2019). In the present study, however, these correlation-based findings cannot establish causal bacteriome–virome interactions or definitive phage–host relationships. These observations suggest that FMT-associated microbial restructuring may extend beyond taxonomic abundance changes to exploratory inter-microbial association patterns, highlighting the importance of ecological organization beyond abundance alone (Mandakovic et al., 2018).

From an experimental and translational perspective, device-based donor conditioning may provide a controlled approach to generate donor fecal material within an intervention-modified intestinal environment rather than relying solely on naturally occurring donor variability. In the present study, DLS was used as a localized duodenal intervention before donor fecal collection, and FMT from these donors was associated with metabolic, intestinal, and microbiome-related changes in recipient GK rats. Nevertheless, because this study did not include an FMT group receiving feces from non-DLS donors, these findings should be interpreted as proof-of-concept evidence for a device–microbiome experimental strategy rather than definitive evidence that DLS specifically enhances FMT efficacy.

In summary, FMT from DLS-conditioned donors was associated with improved glucose tolerance, incretin-related intestinal changes, an increase in HOMA-β within the FMT group, greater pancreatic insulin-positive area at the study endpoint, and coordinated changes in intestinal architecture and microbiome organization in diabetic GK rats. These findings support a hypothesis-generating model in which FMT from DLS-conditioned donors is associated with host metabolic and intestinal phenotypes through post-FMT species-level enrichment or expansion, intestinal remodeling, and altered exploratory bacteriome–virome association patterns. More broadly, this study highlights the potential of combining device-based donor conditioning with microbiota transfer as an experimental strategy to modulate host metabolic phenotypes, while future studies incorporating non-DLS FMT controls, strain-resolved donor–recipient tracking, and mechanistic validation will be required to define DLS-specific, donor-derived, and causal effects.

## Data Availability

The raw shotgun metagenomic sequencing data generated in this study have been deposited in the NCBI Sequence Read Archive under BioProject accession number PRJNA1478298 (https://www.ncbi.nlm.nih.gov/bioproject/PRJNA1478298). Initial preprocessing and host-read prefiltering were performed using a Nextflow workflow composed of standard nf-core modules, including fastp, Cutadapt, Bowtie2, and MultiQC. Bacterial taxonomic profiling was performed using a MetaPhlAn-based Python wrapper script, and viral read-level classification was performed using a custom MEGABLAST-based viral assignment script followed by accession-to-taxid mapping and taxonomic aggregation. To support reproducibility, the study-specific preprocessing parameters, software versions, Nextflow execution files, Python scripts, and downstream R scripts are available in the public GitHub repository: https://github.com/Mynong97/publication-analysis/tree/main/ASAN_FMT_metagenomics_analysis. Additional data supporting the findings of this study are available from the corresponding author upon reasonable request.
