# Supplementary material for: Fecal microbiota transplantation from duodenal light stimulation–conditioned donors is associated with improved glucose tolerance and intestinal incretin-related remodeling in diabetic Goto–Kakizaki rats
Source: Front Cell Infect Microbiol. 2026 Sep 11;16:1900693. doi: 10.3389/fcimb.2026.1900693 (PMC13612201; doi:10.3389/fcimb.2026.1900693)
Supplement: Supplementary file 1 [file DataSheet1.pdf]

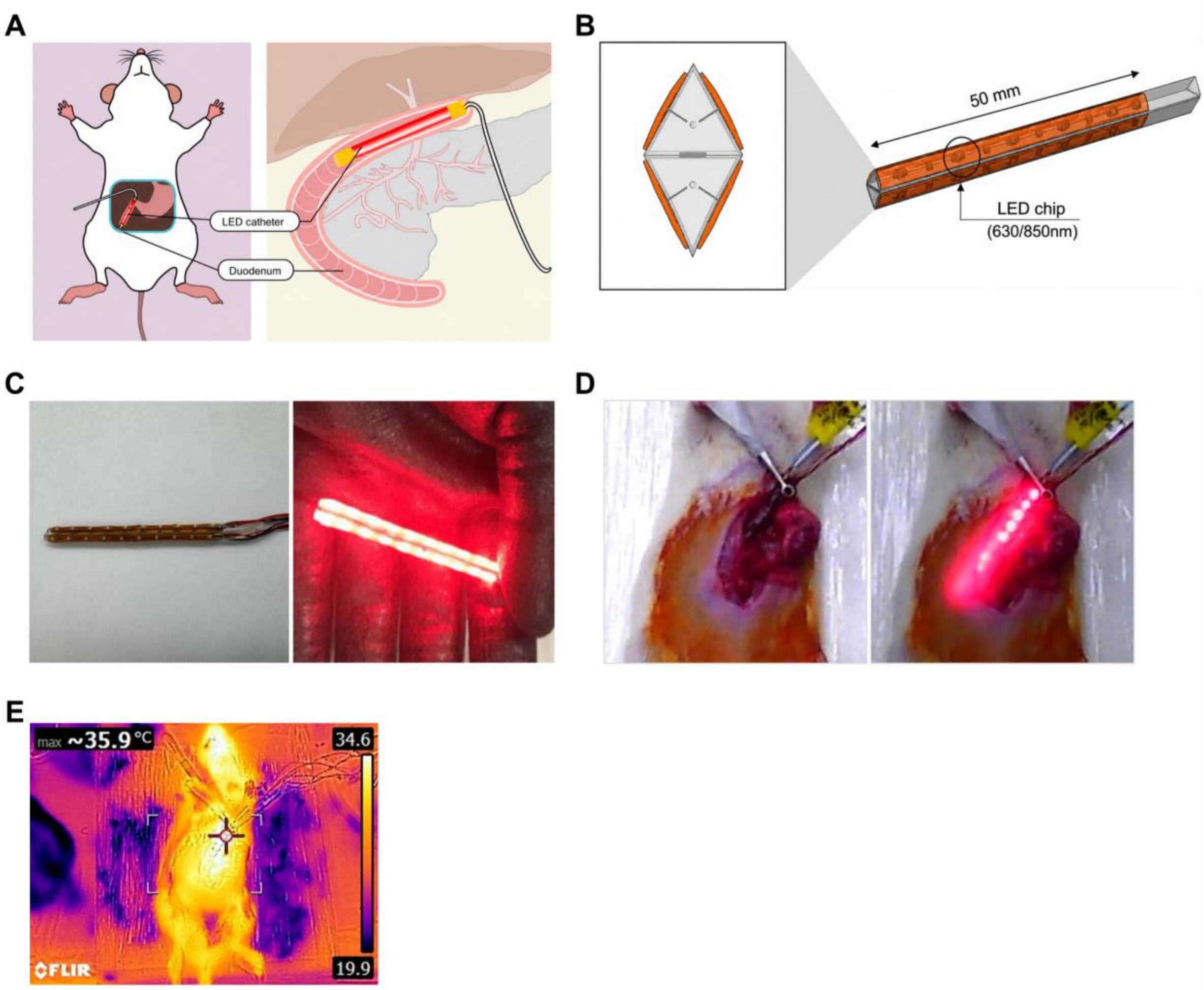

Supplementary Figure 1. Schematic overview and representative images of duodenal light stimulation (DLS) in donor GK rats.

(A) Schematic illustration of the DLS procedure showing insertion and positioning of the LED catheter in the duodenum.

(B) Structural schematic of the dual-wavelength LED catheter showing the four-surface configuration, 50 mm illuminated segment, and 630/850 nm LED chips.

(C) Representative photographs of the LED catheter before and during operation.

(D) Representative intraoperative images before and during LED catheter illumination in the duodenum.

(E) Representative infrared thermal image obtained during DLS for surface temperature monitoring.

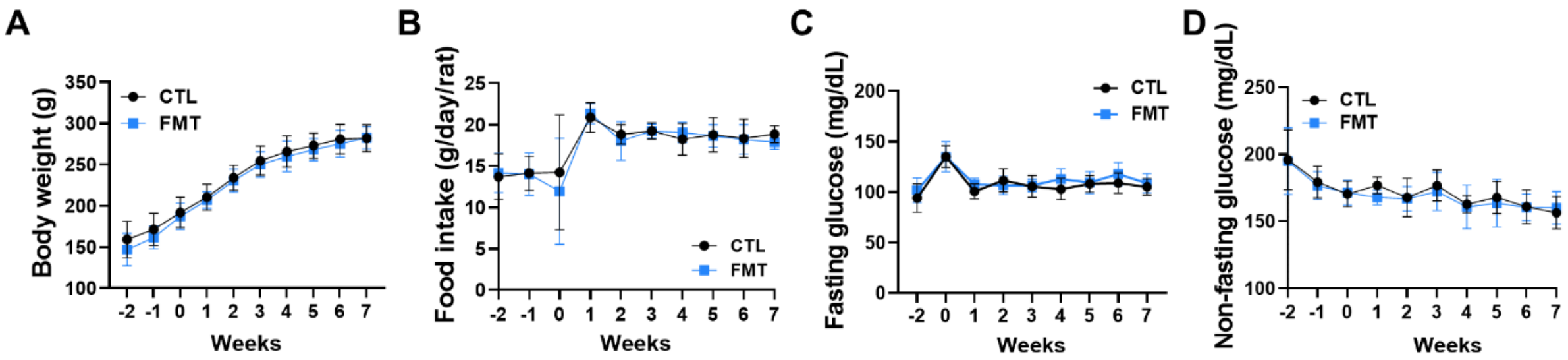

Supplementary Figure 2. General metabolic parameters in recipient GK rats following FMT.

(A) Body weight trajectories of CTL and FMT groups during the experimental period.

(B) Daily food intake.

(C) Fasting blood glucose levels.

(D) Non-fasting blood glucose levels.

Data are presented as mean ± SD (n = 7 per group). Longitudinal data were analyzed using two-way repeated-measures ANOVA with group and week as factors.

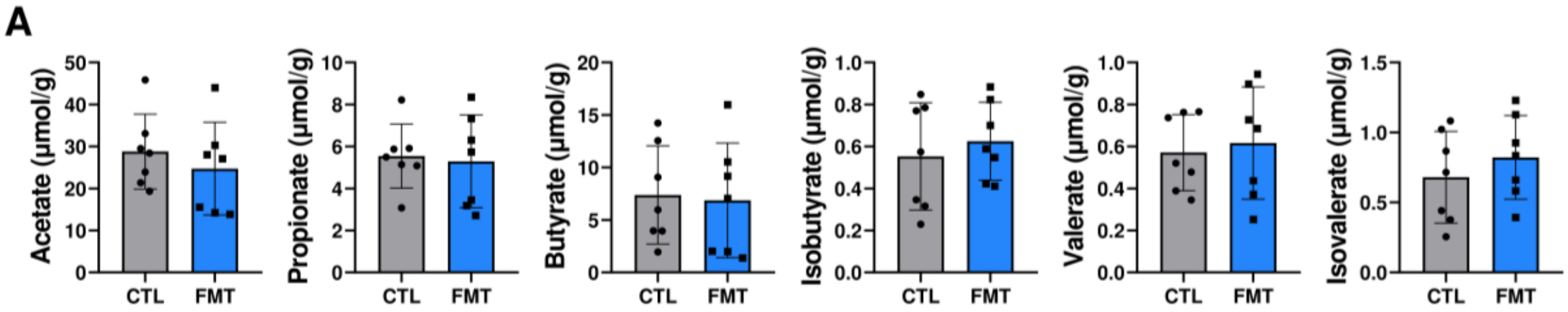

Supplementary Figure 3. Fecal short-chain fatty acid levels in recipient GK rats at Post-Tx.

(A) Fecal short-chain fatty acid (SCFA) levels, including acetate, propionate, butyrate, isobutyrate, valerate, and isovalerate, in CTL and FMT groups at Post-Tx (week 7). SCFA levels are expressed as µmol/g feces.

Data are presented as mean ± SD. Each dot represents an individual animal (n = 7 per group). Statistical comparisons between CTL and FMT groups were performed using Welch's unpaired two-tailed t-test for each SCFA.

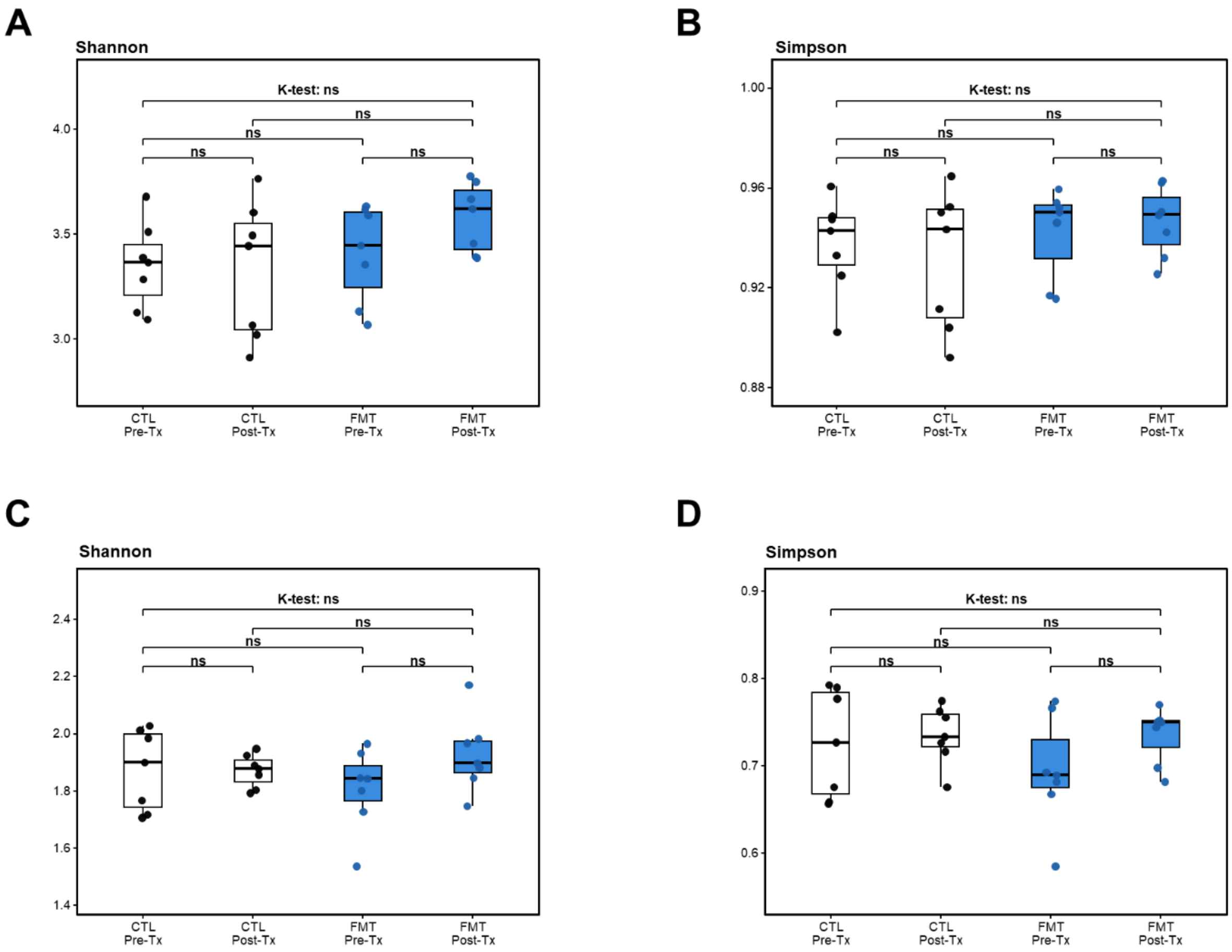

Supplementary Figure 4. α-diversity analysis of bacteriome and virome profiles in recipient GK rats.

(A) Bacteriome Shannon diversity in CTL and FMT groups at Pre-Tx and Post-Tx.

(B) Bacteriome Simpson diversity in CTL and FMT groups at Pre-Tx and Post-Tx.

(C) Virome Shannon diversity in CTL and FMT groups at Pre-Tx and Post-Tx.

(D) Virome Simpson diversity in CTL and FMT groups at Pre-Tx and Post-Tx.

Data are presented as individual values with mean ± SD (n = 7 per group). Between-group comparisons at each time point were performed using the Mann–Whitney U test, and within-group longitudinal comparisons were analyzed using the Wilcoxon signed-rank test. Overall group differences across the four conditions were assessed using the Kruskal–Wallis test. Pre-Tx corresponds to week −2 before FMT, and Post-Tx corresponds to week 7.

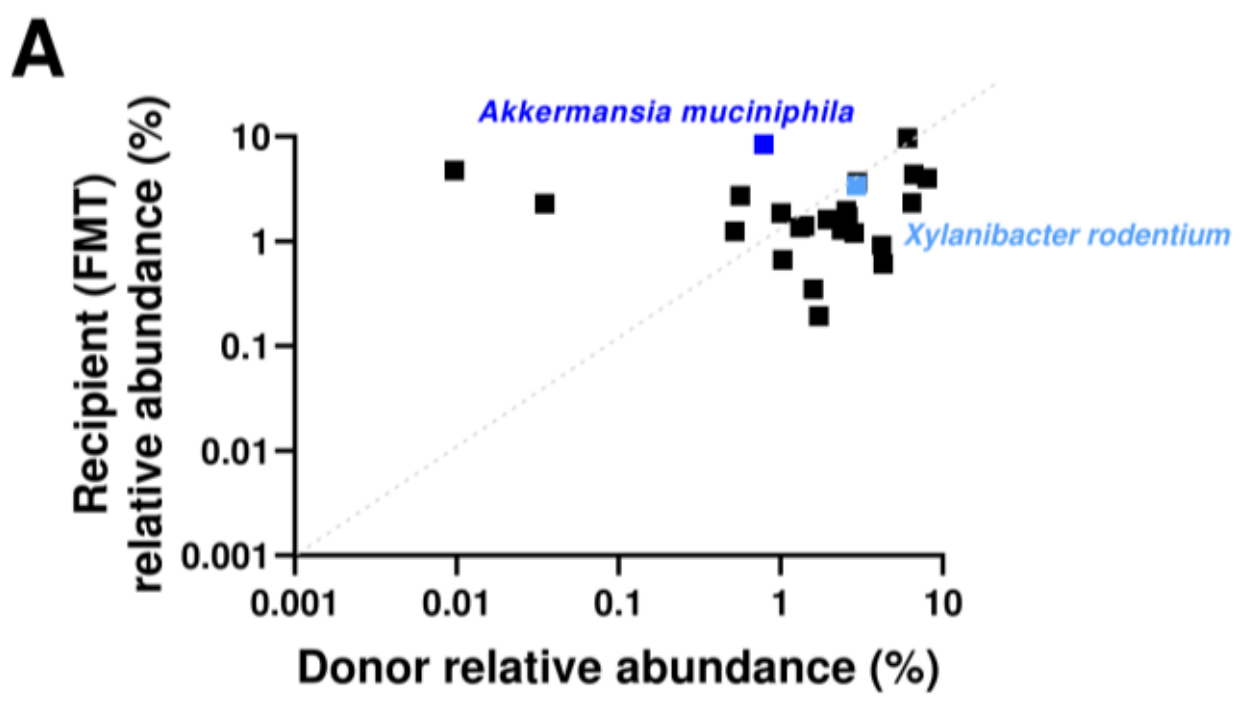

Supplementary Figure 5. Descriptive species-level comparison between donor fecal samples and FMT recipient samples at Post-Tx.

Scatter plot comparing the relative abundance of bacterial species detected in donor fecal samples and FMT recipient fecal samples at Post-Tx (week 7). Each point represents a bacterial species. The dashed diagonal line indicates equal relative abundance between donor and FMT recipient samples. Taxa above the diagonal show higher relative abundance in FMT recipients, whereas taxa below the diagonal show lower relative abundance in FMT recipients. This species-level comparison was performed as a descriptive analysis and was not used to infer donor-derived engraftment, microbial transmission, or strain-level donor origin.
